# Microbiome Taxonomic and Functional Differences in C3H/HeJ Mice Fed a Long-Term High-Fat Diet with Beef Protein ± Ammonium Hydroxide Supplementation

**DOI:** 10.3390/nu16111613

**Published:** 2024-05-25

**Authors:** Emily C. Garrison, Amanda M. V. Brown, McKinlee M. Salazar, Benjamin Barr, Naima Moustaid-Moussa, Lauren S. Gollahon

**Affiliations:** 1 Department of Biological Sciences, Texas Tech University, 2500 Broadway, Lubbock, TX 79409, USA; garrisoncemily@gmail.com (E.C.G.); amanda.mv.brown@ttu.edu (A.M.V.B.); mckinlee.salazar@ttu.edu (M.M.S.); benjamin.barr@ttu.edu (B.B.); 2 Department of Nutritional Sciences, Texas Tech University, 2500 Broadway, Lubbock, TX 79409, USA; naima.moustaid-moussa@ttu.edu; 3 Obesity Research Institute, Texas Tech University, 2500 Broadway, Lubbock, TX 79409, USA

**Keywords:** dietary beef protein, ammonium supplementation, obesity, microbiome changes

## Abstract

Studies have suggested that alkalinized foods may reduce the effects of the acidogenic Western diet in promoting obesity, metabolic syndrome, type 2 diabetes, cancer, and coronary heart disease. Indeed, a recent study in mice fed a high-fat diet containing dietary beef supplemented with ammonium hydroxide showed improvement in a suite of metabolic outcomes. However, the effects of dietary protein ammonium supplementation on the microbiome remain unknown. In this study, the effects of ammonium supplementation on beef protein towards microbiome taxa and function in a high-fat diet were analyzed. Fecal microbiomes were characterized using a shotgun metagenomic approach for 16-month-old male and female mice after long-term diet treatments. The results for ammoniated diets showed that several bacteria known to be associated with health benefits increased significantly, including *Romboutsia*, *Oscillospiraceae*, and *Lactococcus cremoris*. The beneficial mucin-degrader *Akkermansia* was especially abundant, with a high prevalence (~86%) in females. Concurrently, the phyla Actinomycetota (Actinobacteria) and Bacteroidota (Bacteroidetes) were significantly reduced. While sex was a confounding factor affecting microbiome responses to ammonium supplementation in dietary protein, it is worth noting that several putatively beneficial microbiome functions increased with ammonium supplementation, such as glycine betaine transport, xenobiotic detoxification, enhanced defense, and others. Conversely, many disease-associated microbiome functions reduced. Importantly, modifying protein pH alone via ammonium supplementation induced beneficial microbiota changes. Taken together, these results suggest that ammonium-supplemented proteins may mediate some negative microbiome-associated effects of high-fat/Western diets.

## 1. Introduction

In recent years, “alkalinized” foods that reduce the effects of the acidogenic “Western diet” [1] have received attention for their potential to reduce the risk or pathogenesis of many diseases [2,3,4,5]. However, to date, the microbiome’s role in such benefits has been poorly characterized. Mechanistically, the health impacts are thought to result from low-grade metabolic acidosis, characterized by a slight decrease in blood pH from the normal range [6,7,8,9]. This slight blood acidosis has been linked to increased potential renal acid load (PRAL) and negative health effects ranging from kidney and bone issues to metabolic disorders, cancers, diabetes, and others through complex acid–base processes in the body (for review, see Williamson et al., 2021 [2]), ultimately initiated by the breakdown of protein-rich foods in the diet. Addressing the question of how the general Western diet—rich in saturated fats, proteins derived from processed meats, refined grains, alcohol, and salt, and low in fruits and vegetable fibers—and the diet’s acidogenic properties contribute to adverse health outcomes is complex.

Although numerous studies show that both the microbiome and diet are tied to major diseases, it remains unclear how the microbiome is involved in response to acidogenic diets. For example, obesity and its comorbidities, including coronary heart disease (CHD), type 2 diabetes (T2D), stroke, metabolic syndrome (MetS), and certain cancers [1,10,11,12,13], are linked to a high-fat, low-fiber diet [14]. All these factors are typically intimately tied to microbiome changes that either result from these health conditions or contribute to them. But a few studies have examined how dietary pH shifts change microbiomes in ways that are relevant to the acidogenic effects of Western diets. For example, Ilhan et al. (2017) [15] tested the effects of modified pH on lactate-producing microbiota and their microbiomes. They reported that pH is the strongest driver of microbial community structure and function, and microbial and metabolic interactions [15]. Another study tested animal microbiome changes resulting from ammonia exposure and found significant changes in the growth of common microbiome taxa [16]. Firrman et al. (2022) [17] showed that raising the pH of the human gut microbiota in vitro increased microbiome diversity and beneficial short-chain fatty acids (SCFAs).

Given the importance of dietary proteins, there has been growing interest in ways to modify pH in meats to improve their health properties [18]. While a high-protein diet alone can negate some effects of obesity and reduce fat mass without significant weight loss [19], when combined with other components of the Western diet (saturated fats, simple sugars, etc.), meat protein can lead to adverse health effects [20]. According to the United States Department of Agriculture, in 2017, Americans consumed close to 56 lbs of beef per person per year [21]. A recent study examined whether the effects of modifying pH in dietary beef protein prior to complete diet formulation, fed to mice reared on a high-fat diet, could attenuate some of the pathological effects associated with the acidogenic Western diet [5]. Modification of beef pH was performed through an ammoniation process that is USDA-approved in industrial use and was therefore deemed a practical way to implement this change in this study [2].

Although the recent ammoniated high-fat beef diet study [5] produced remarkable differences among experimental groups, reducing adiposity and body weight and improving glucose clearance, it did not assess the microbiome’s role. Therefore, the aim of the current study was to assess microbiome taxonomic and functional changes associated with ammonium-supplemented beef in mice fed high-fat diets using shotgun metagenomic sequence analysis. These results are presented by first demonstrating the effects of diets ± ammonium supplementation on mouse weight gain and survivorship. Since this was one of the first studies to analyze CH3/heJ male and female mice fed high-fat diets with beef protein ± ammonium supplementation over a long term, a shotgun metagenomics approach was performed over 16S amplicons to generate a more in-depth understanding of changes in the microbiota [22]. Following this, the results describe sequence yield, assembly features, and annotated genes to demonstrate the quality of the output for further analysis. The results were then described in phylum-level and lower-level taxonomic classifications of the refined bins. From this point forward, a number of statistical tests of differentially abundant taxa from the yields were performed to report both overt and subtle changes in microbial species on aged mice fed high-fat diets with beef protein ± ammonium supplementation. We found significant shifts in the microbiota of the mice that consumed the ammonium-supplemented beef protein diet compared to those consuming a ‘standard’ beef protein diet, suggesting that ammonium-supplemented foods may contribute to health benefits for those eating high-fat or Western diets.

## 2. Methods

### 2.1. Mouse Study Design

The mice used in this specific study were part of a much larger study under Texas Tech University IACUC protocol 19021-02. For the study, C3H/HeJ mice (*Mus musculus*) developed by Jackson Laboratories (JAX stock #000659) were used. At all times, mouse care and handling were followed as per the protocol. The strain of mice selected was a robust, generic strain, unmodified for disease susceptibility or resistance and commonly used in cancer, inflammation, immunology, and cardiovascular studies. For this specific study, a total of 32 C3H/HeJ mice (16 females and 16 males) were housed in cages containing 4 mice per cage and 2 cages per diet type per sex. The mice arrived at 4 weeks old, were acclimated for 2 weeks, and started their respective diets at 6 weeks with equal numbers of male and female mice randomly assigned to either an ammonium-supplemented high-fat beef diet (HFBN) or an unsupplemented, high-fat beef diet (HFB) (served as control). The mice were maintained on this diet for the duration of the study, with fecal material for microbiome analysis collected at 16 months. Sixteen months was selected as the time point for fecal sampling and microbiome analysis, as individual viability decreased significantly approaching the 18-month mark, and it was unknown whether the sample size would become too low by then. Dietary macromolecular distribution as kcal% was 18% protein, 46% fat, and 36% carbohydrates. The total dietary components and formulations are listed in Table 1. The mice were housed in a ventilated cage with a 12 h light/dark cycle at 22–23 °C and 70% humidity. They also had access to their respective diets and water ad libitum. Their weight and food intake were measured weekly. At the end of the dietary intervention period (18 months for this study), the mice were fasted for 2 h before euthanasia.

### 2.2. Diet Preparation and Composition

The diets were prepared by Research Diets, Inc. (New Brunswick, NJ, USA). Both diets were created using ground beef supplied by Empirical Foods, Inc. (North Sioux, SD, USA). For the supplemented diet, ammonium hydroxide was added to the raw ground beef product, generating a pH of ~9.5, cooked, freeze-dried, and sent to Research Diets Inc. for final mouse chow preparation. For the unsupplemented diet, beef was cooked and freeze-dried in the same manner without ammonium hydroxide. At this point, all other dietary components were added such that the total beef content by weight was about 38% and the overall protein, carbohydrate, and fat compositions were equal in both diets. Micronutrients and nutritional analysis were provided by Eurofins Scientific Inc. (Des Moines, IA, USA). The ammonium-supplemented high-fat beef diet is hereafter denoted as HFBN, and the control high-fat beef diet (no supplementation) is hereafter denoted as HFB. To determine the final pH of each diet, food pellets were homogenized with Milli-Q^®^ water (Millipore Sigma, Boston, MA, USA), and tested in triplicate using a benchtop pH meter. The diets differed in pH: the HFBN diet had a pH of ~8.256, while the HFB diet had a pH of ~7.343. In all other aspects, the diet compositions were similar (Table 1). The diets were stored in a −20 °C freezer until use.

### 2.3. Mouse Metrics

The mice were weighed weekly and examined for any physical abnormalities throughout the study. These masses were used to generate trends for total mass change over time and taken into consideration when assessing the overall health of each group. Locally estimated scatterplot smoothing (LOESS) was used to generate trendlines for changes in total mass associated with age. Food pellets (40 g/mouse) were also replaced weekly, and food consumption was recorded. Based on the consistency of consumption, this is summarized in Table 2. Along with weekly measurements, survivability was tracked and assessed using Kaplan–Meier analysis for adverse events. This analysis was performed in R using the *survival* package (Version 3.4-6), and significance was assessed using the log-rank test (Mantel–Haenszel).

At the time of microbiome collection, mouse weights were recorded for the females (HFBN-F, HFB-F) and the males of each treatment (HFBN-M, HFB-M) (Gollahon—unpublished data, manuscripts in preparation).

### 2.4. Fecal Collection and Processing

At age 16 months, the mice were removed from their home cages and placed into an empty sterile cage, where they remained for ~45 min. Stool samples were collected as they were produced. The mice were then placed back into their home cages and returned to their housing area. The stool belonging to both cages from the cohort (i.e., HFBN-F, both cages) was pooled into one sample (i.e., HFBN-F) to obtain the averages of the community following the methodology described previously [23,24,25]. Fecal samples were collected in a sterile 2 mL tube, placed directly on ice, and then transferred to −80 °C. The samples were sent to Azenta (South Plainfield, NJ, USA) for DNA extraction and whole-genome shotgun metagenomic sequencing using Illumina (San Diego, CA, USA) with 150 bp PE sequencing.

### 2.5. Metagenomic Assembly and Initial Taxonomic Analysis

Initial analyses were performed to obtain quality taxonomically identified metagenomes. The basic pipeline involved genome assembly and taxonomic binning using the metaWRAP [26] software pipeline. Briefly, this involved pre-processing with the ReadQC module using PEAR (Current Tag: 0.9.11) [27] and Trimmomatic (version 0.40) [28] for merging and trimming reads. The reads were then assembled using metaSPAdes [29]. Assembly contigs were then taxonomically binned using the metaWRAP-Binning module, which includes CONCOCT, MaxBin2, and metaBAT2 [30,31,32]. Next, quality bins were extracted using the Bin_Refinement module with stringent parameters (90% minimum completion and 10% maximum contamination). The refined bins were then run through the Reassemble_Bins module, which maps reads to the individual bins and reassembles bins via SPAdes [33] and assesses the final results using CheckM [34]. Once clean and quality bins were obtained, the metaWRAP Classify_Bins module was used to investigate the taxonomic composition of each bin. Bacterial taxonomy and abundance were calculated on the reads mapping to these polished bins using Centrifuge [35], which uses an indexing scheme based on the Burrows–Wheeler transform (BWT) index, as well as the Ferragina–Manzini (FM) index.

Blobology [36] was used to create abundance–GC plots of the initial bins by analyzing the GC content and read coverage, as well as matching the sequences to the NCBI database using BLAST+ [37]. To create the stacked bar plot of relative phyla abundance, the R package tidyverse (Version 1.3.0) [38] was used in combination with the R package RColorBrewer. Kronagrams depicting relative taxonomic abundance were created with the outputs of Centrifuge using Krona (Version 3.9) [39].

### 2.6. Refined Taxonomic Analysis and Differential Abundance Tests

Although the taxonomic binning described above was well suited for characterizing abundant species and strains, we also performed additional analyses using MetaPhlAn4 [40,41] to improve the characterization of previously uncharacterized taxa-like strains and species (i.e., species-level genome bins, or SGBs), provide additional resolution of higher taxonomic groups, and allow annotation for taxa with more fragmented or incomplete genomes compared to the pipeline above [42]. For MetaPhlAn analyses, the full set of trimmed reads (prior to binning) were used, mapping to the mpa database vOct22 of CHOCOPhlAnSGB 202212. Then, the outputs were merged into a combined sample table to generate a Bray–Curtis correlation dendrogram and heatmap using hclust2.py from the MetaPhlAn suite, and Bray–Curtis dissimilarity was calculated using the calculate.diversity.R script.

To assess the beta diversity between groups of sample taxa and determine statistical differences in taxonomic abundance, MetaPhlAn taxonomic abundance tables were converted into phyloseq-class objects for analysis using the R packages phyloseq [43] and ggplot2. Bray–Curtis dissimilarity was computed between groups using the vegan::adonis2 R package [44], differences were assessed using PERMANOVA, and species-level genome bins (SGBs) and sample groups were plotted using the phyloseq function ‘ordinate’. Analysis of Compositions of Microbiomes with Bias Correction 2 (ANCOM-BC2) [45,46] was used to perform differential abundance (DA) analysis of the taxa for each treatment group. This software addresses the problem of compositionality and then assesses and corrects structural zeros (i.e., missing taxa) that significantly impact microbiome DA analyses. ANCOM-BC2 then performs a sensitivity analysis on the zero-corrected pseudo-counts, applies linear regression models to each taxon’s bias-corrected abundance, and performs corrections for multiple testing. We used the parameters ‘p_adj_method’ and ‘fdr’ to perform multiple testing corrections (*p*-values converted to q-values) and parameters ‘fix_formula = “Sex + Group”’ to assess the fixed effects of each metadata type on the q-value (i.e., interactions among factors).

### 2.7. Functional Analysis and Gene Ontology Enrichment Tests

Gene annotation was performed on the initial assemblies generated above (prior to binning) using Prokka [47]. The detection of ortholog clusters for pangenomic analysis was performed using Roary [48]. Venn diagrams were created via an online drawing tool found at http://bioinformatics.psb.ugent.be/webtools/Venn/ (accessed on 12 May 2022), and selected unshared gene sets were compared to the shared or ‘universal’ gene sets in topGO (accessed on 14 May 2022) [49], which tested for differences in microbiome functional profiles among samples using gene ontology (GO) enrichment analysis, with the ‘weight01.fisher’ statistic. To prepare annotations for topGO, a list of GO ID-to-gene mappings ‘go_annot’ was created by downloading UniProtKB database GO terms for abundant taxa in the metapangenomes microbiomes, and missing genes or synonyms were resolved by cross-referencing with MetaCyc/KEGG. TopGO was performed rapidly using the R script aip_topgo_usage.consider_universe.R (https://github.com/lyijin/topGO_pipeline/) (accessed on 20 May 2022). Semantic space plots of gene ontology enrichment outputs from topGO were created using ‘Go-Figure!’ [50].

## 3. Results

### 3.1. Survivability Assessment

**Figure 1:**
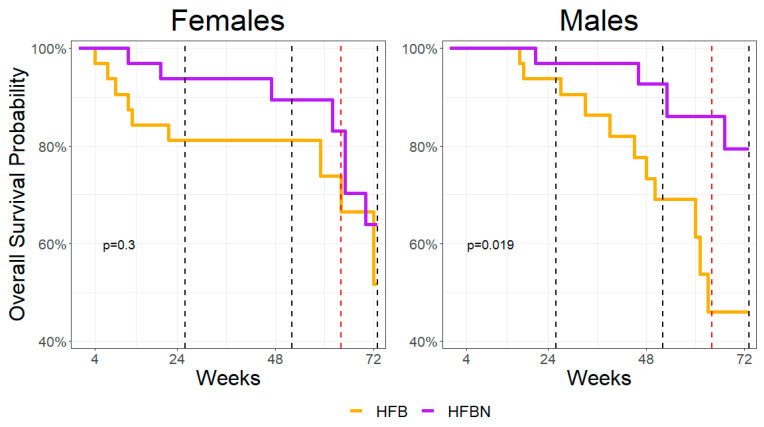
Kaplan–Meier survivability assessment over 72 weeks (18 months) and sex-specific comparison of each diet (**Left**: females; **right**: males). The black dashed lines indicate terminal censorship points for tissue collection used for cross-sectional analysis of organ function. The red dashed line indicates the initiation of fecal sampling at week 64 (16 months). The curves are compared using the log-rank test, with α = 0.05 and *df* = 1.

Kaplan–Meier analysis was used to determine survivability as a function of aging, and comparisons were performed between each group (Figure 1). The *p*-values shown are based on comparisons within each sex. A comparison was performed at *n* = 32, α = 0.05, and degrees of freedom = 1. The chi-square values were 1.2 for females and 5.5 for males. The dramatic decrease in survivability leading up to 16 months (64 weeks) was the rationale for initiating the microbiome assessment at that point instead of 18 months because of the possibility of significantly decreased sample sizes.

### 3.2. Food Consumption and Mass

The mice consumed similar amounts of food, regardless of treatment, with major differences attributable to sex. Table 2 details the average amount of food consumed per mouse per week.

**Figure 2:**
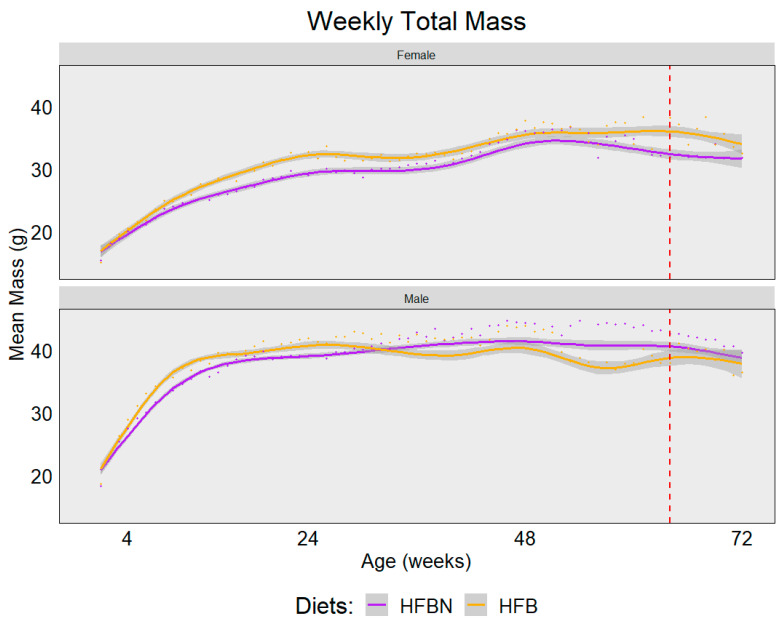
Locally estimated scatterplot smoothing (LOESS) model showing trends for change in mass over time separated by sex. The red dashed line indicates the fecal sampling time point, 64 weeks (16 months), and the gray area indicates the 95% confidence interval of the LOESS line. + = starting point for study.

The weekly trend in mass change is depicted using a locally estimated scatterplot smoothing (LOESS) model (Figure 2). There appears to be little difference in the trend between the female groups with the exception of HFBN-F, which maintained lower mass than HFB-F. Male mass is consistently elevated compared with female mass for both groups. Interestingly, HFBN-M maintained a more consistent mass throughout the course of the study compared with HFB-M. Upon fecal sampling at 16 months, the average individual weights were: HFBN-F = 32 g; HFBN-M = 42.6 g; HFB-F = 38.2 g; and HFB-M = 38 g.

### 3.3. Sequence Yield, Assembly Features, and Annotated Genes

The read output was of high quality, with similar yield across samples (Table 3). Initial assemblies, before binning and filtering, produced between 180 and 343 thousand contigs with contig lengths of up to 684,229 bp and total assembled contig lengths between 173 and 323 million bp (Table 3). The assembly N50 values were similar across the samples and above 2300 bp. The number of genes annotated per sample from these initial assemblies was between 176 and 326 thousand, and these values correlated with the total contig length (Table 3); however, the majority of annotated genes had no known function (i.e., annotated as ‘hypothetical protein’) or occurred as ortholog/paralog sets within and among genomes, such that the number of unique gene orthologs with known function was between 3930 and 4359 genes per sample (Table 3).

### 3.4. Phylum-Level Taxonomic Composition of metaWRAP-Refined Bins

Based on Centrifuge software analyses, at the level of the phylum, Verrucomicrobiota (synonym Verrucomicrobia) were highly abundant across all samples (Supplementary Figure S1). Ammoniated HF beef diet females (HFBN-F) had the greatest abundance at 86% Verrucomicrobiota for classified bins, and control females HFB-F had 64% Verrucomicrobiota, followed by control males HFB-M at 60% and ammonium-supplemented males HFBN-M at 56%. Across all samples, the phylum Bacillota (synonym Firmicutes) was the next most abundant, with males having higher abundances than females. HFBN-M showed a 25% abundance of Firmicutes, with HFBN-F having just 4%. Pseudomonadota (synonym Proteobacteria) were the next most abundant bacteria overall, with slightly increased proportions in controls (unammoniated beef), while Actinomycetota (synonym Actinobacteria) and Bacteroidota (synonym Bacteroidetes) showed a similar pattern, being slightly more abundant in controls.

### 3.5. Lower-Level Taxonomic Classification of metaWRAP-Refined Bins

**Figure 3:**
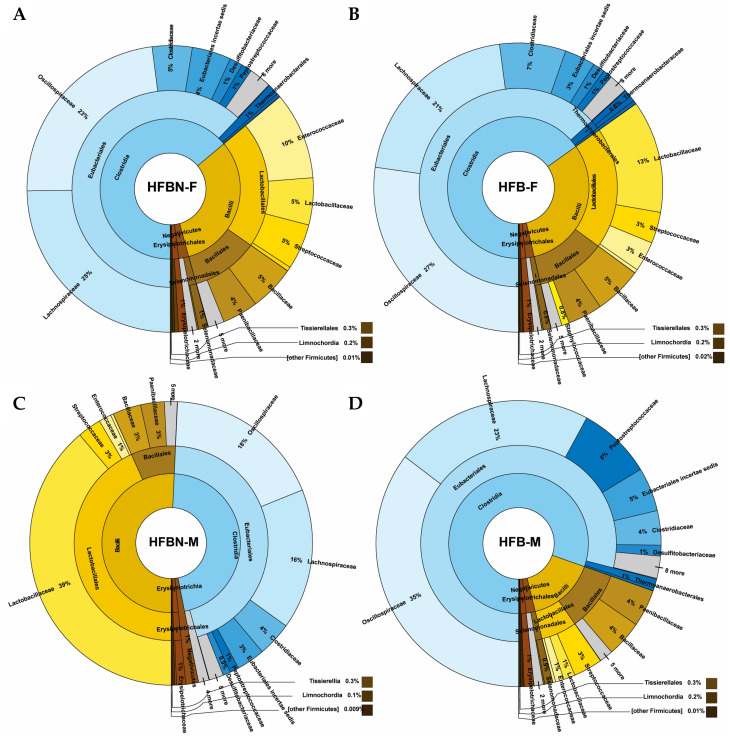
Taxonomic profiles created from the binned quality metagenomes, depicted with Krona charts, for Bacillota (Firmicutes). (**A**) HFB-F, Group 3 females; (**B**) HFBN-F, Group 4 females; (**C**) HFB-M, Group 3 males; and (**D**) HFBN-M, Group 4 males.

**Figure 4:**
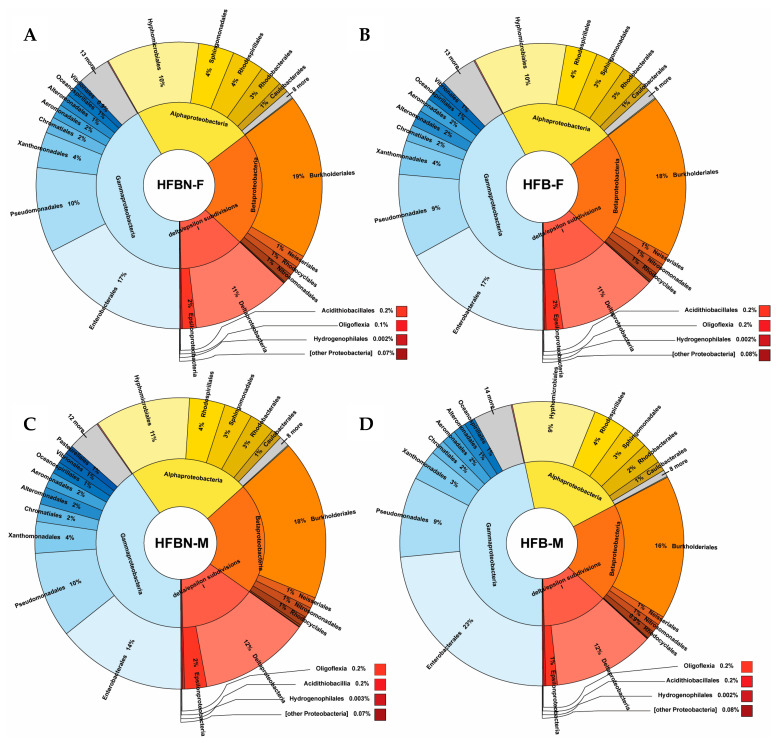
Taxonomic profiles created from the binned quality metagenomes, depicted with Krona charts, for Pseudomonadota (Proteobacteria). (**A**) HFB-F, Group 3 females; (**B**) HFBN-F, Group 4 females; (**C**) HFB-M, Group 3 males; and (**D**) HFBN-M, Group 4 males.

**Figure 5:**
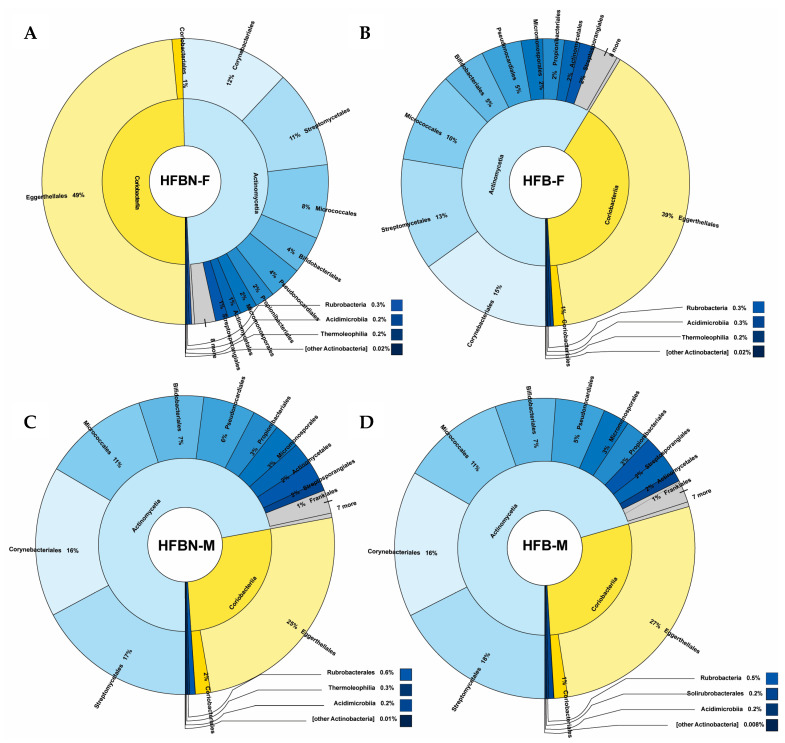
Taxonomic profiles created from the binned quality metagenomes, depicted with Krona charts, for Actinomycetota (Actinobacteria). (**A**) HFB-F, Group 3 females; (**B**) HFBN-F, Group 4 females; (**C**) HFB-M, Group 3 males; and (**D**) HFBN-M, Group 4 males.

**Figure 6:**
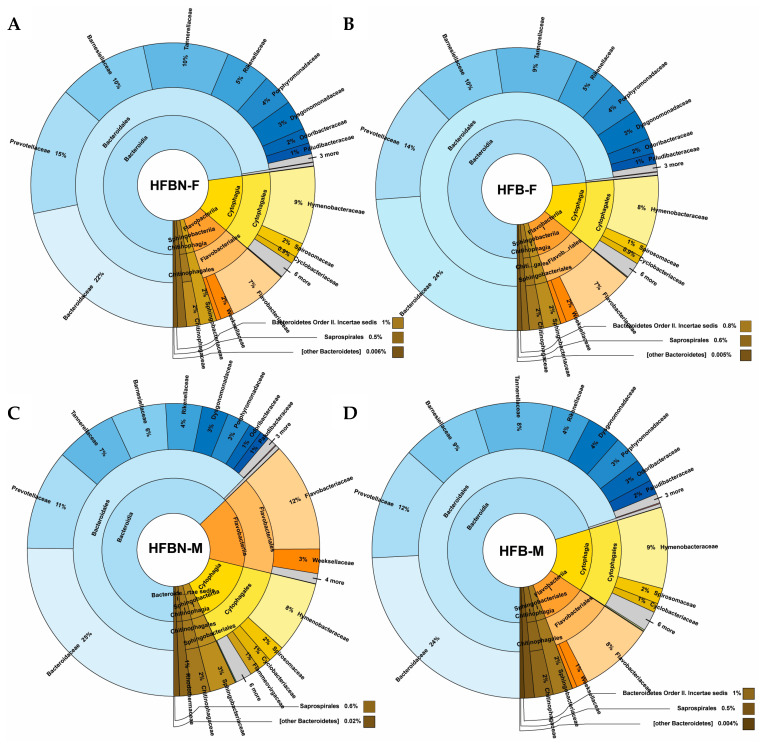
Taxonomic profiles created from the binned quality metagenomes, depicted with Krona charts, for Bacteroidota (Bacteroidetes). (**A**) HFB-F, Group 3 females; (**B**) HFBN-F, Group 4 females; (**C**) HFB-M, Group 3 males; and (**D**) HFBN-M, Group 4 males.

Initial binning produced abundance–GC plots that revealed similar clusters across samples (Supplementary Figure S2), with class-level taxonomic groupings showing Lactobacillales notably abundant in HFBN-M, and males of both groups displaying more Bacillales than female groups. The dominant phylum Verrucomicrobiota was made up of the single species *Akkermansia muciniphila*. For assembled polished binned taxa classified using the metaWRAP pipeline, the next four abundant phyla (Firmicutes, Proteobacteria, Actinobacteria, and Bacteroidetes) showed proportional differences among samples (Figure 3, Figure 4, Figure 5 and Figure 6). For example, among the Firmicutes (Figure 3), Clostridia were more abundant than Bacilli, except in HFBN-M, which had a larger relative abundance of Bacilli. HFB-M had a large portion of Clostridia (80% of all Firmicutes). Eubacteriales was the dominant order across all groups, with *Lachnospiraceae* and *Oscillospiraceae* being the most abundant families. *Oscillospiraceae* was relatively abundant in all groups except HFBN-F, which had a high relative abundance of *Lachnospiraceae.* Within this family, at the genus level, all groups demonstrated *Lachnoclostridium* as a highly prevalent genus, followed by *Roseburia*. The genus *Flavonifractor* was dominant within the *Oscillospiraceae*.

Within the Bacilli (Figure 3), HFBN-F, HFBN-M, and HFB-F had a high relative abundance of Lactobacillales compared to Bacillales. HFB-M demonstrated the opposite pattern, with 63% of Bacilli being of the order Bacillales compared to less than 36% in the other groups. At the family level, HFB-M also had a high relative abundance of *Paenibacillaceae* compared to *Bacillaceae*, which was the opposite of the other groups. *Lactobacillaceae* was relatively the most abundant family in HFBN-M and HFB-F, whereas *Enterococcaceae* was most abundant in HFBN-F, and *Streptococcaceae* was most abundant in HFB-M.

Among Proteobacteria taxa (Figure 4) in female mice, HFBN-F and HFB-F were similar for groups such as Pseudomondales, Burkholderiales, and Enterobacterales. The male ammoniated beef diet mice (HFBN-M) demonstrated low relative abundance of Enterobacterales. In contrast, the male mice on unammoniated beef diets (HFB-M) displayed high relative abundances of Enterobacterales.

Actinobacteria were dominated by Actinomycetia and Coriobacteriia (Figure 5), with the male groups having slightly more Actinomycetia, while the females HFB-F and HFBN-F had less of this taxon. Eggerthellales were the dominant Coriobacteriia across all samples. The male mice had relatively high Streptomycetales, while the females had higher Corynebacteriales. Micrococcales and Bifidobacteriales were at low abundance across all groups.

Bacteroidetes (Figure 6) comprised only a small portion of the total microbiome, with *Bacteroidaceae* being a relatively abundant family, followed by *Prevotellaceae*. Cytophagia and Flavobacteriia were relatively abundant in all groups except HFBN-M.

### 3.6. Refined Taxonomic Data Classification Using MetaPhlAn4

**Figure 7:**
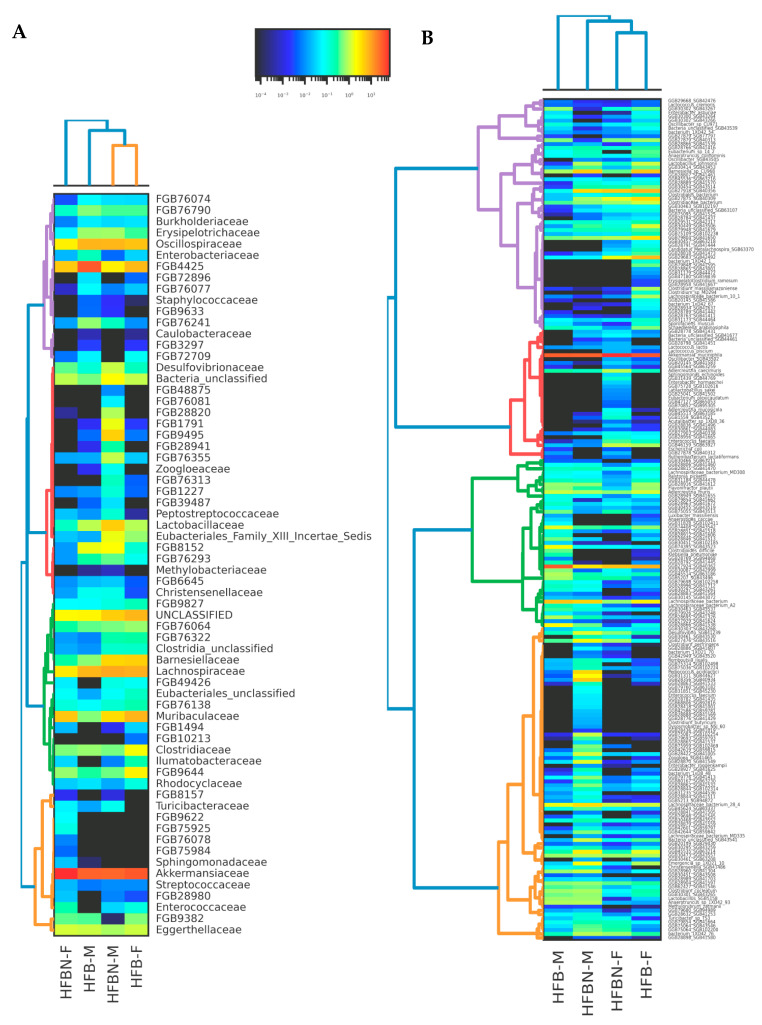
Relative abundance Bray–Curtis dendrogram heatmaps of microbiome taxonomic bins. (**A**) Family level—60 most abundant families; (**B**) species level—220 most abundant species, where MetaPhlAn4-based unclassified taxon bin-denoted names “FGB” and “SGB” represent clustered sequence genomic bins for mice fed on HF beef diets + ammoniation (HFBN-F, females; HFBN-M, males) or unsupplemented diets (HFB-F, females; HFB-M, males). Note: Individual species in B can be viewed by using the zoom tool.

**Figure 8:**
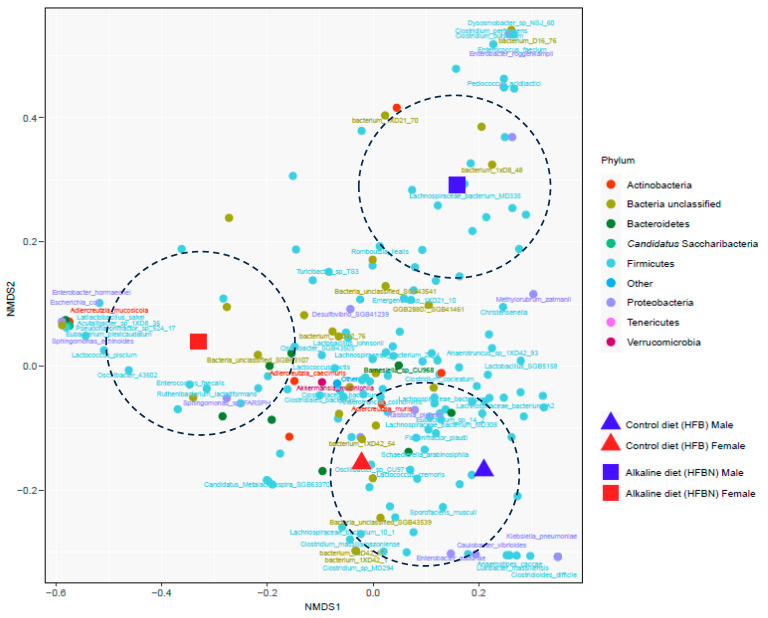
Divergence of taxa (species-level groups, colored by phyla) and treatment groups depicted on an NMDS ordination plot for microbiomes. Results from mice fed on high-fat beef diets with ammoniation (HFBN-F, females; HFBN-M, males) or unsupplemented diets (HFB-F, females; HFB-M, males). For ease of display, taxon names are indicated only for groups that could be assigned to the taxon names described. Note: Individual species can be viewed using the zoom tool.

Refined taxonomic classification using the MetaPhlAn4 pipeline produced 792 taxonomic groups across these samples, with 448 taxa defined to genus level and 232 taxa defined to species or strain level. Taxonomic comparisons of families and species, clustered using Bray–Curtis dissimilarity, revealed variable patterns (Figure 7), with many taxa not significantly different in abundance based on just diet treatment or just sex at either the family level (Figure 7A) or species level (Figure 7B). However, microbiomes showed divergence affected by both diet and sex, as can be seen in the NMDS ordination plots at the species level (Figure 8), in which the controls (HFB-M and HFB-F) are grouped more closely together than treatments with ammonium-supplemented diets (HFBN-F and HFBN-M). However, PERMANOVA results for the variables sex and diet treatment across all microbes together were non-significant.

### 3.7. Statistical Tests of Differentially Abundant Taxa from ANCOM-BC2

Despite the lack of significance in PERMANOVA tests comparing diets or sex across all microbiota, individual taxa at the phylum level, genus level, and species level were found to be significantly different among the groups in ANCOM-BC2 analyses. For example, at the phylum level, testing the seven groups Firmicutes, Actinobacteria, Verrucomicrobiota, Bacteroidetes, Proteobacteria, ‘Other phyla’, and ‘Bacteria unclassified’, there were statistically significant differences in abundances of taxa among groups. Actinobacteria (q = 0.01274678) and Bacteroidetes (q = 0.01274678) were less abundant in ammoniated diets, regardless of sex. There were statistically significant sex effects, with males having significantly lower levels of ‘Other phyla’ (q = 3.027457 × 10^−8^) in both diet groups.

At the genus level, 4 out of 77 taxa were significantly differentially abundant, based on ANCOM-BC2 analyses. One undetermined genus of Firmicutes (GGB28851) was lower in mice fed with ammonium-supplemented diets (q = 7.92629 × 10^−6^), and three other Firmicutes genera were more abundant in ammonium-supplemented diets, including *Romboutsia* (a Clostridia in the family *Peptostreptococcaceae*) (q = 0.008752648), a genus of Clostridia in the family *Oscillospiraceae* (GBB75064) (q = 2.09731 × 10^−7^), and an undetermined Firmicutes (GGB45624) (q = 2.899739 × 10^−5^). At the genus level, six taxa were also significantly differentially abundant. Of these taxa, three were more abundant in the females, including two genera of Bacteroidetes in the family *Muribaculaceae*, GGB27903 (q = 0.04249347) and GBB46159 (q = 9.147622 × 10^−6^), and one Firmicutes (a Clostridia in the family *Peptostreptococcaceae*) (GGB29683) (q = 5.741956 × 10^−5^). The three more abundant in the males included undetermined Firmicutes GGB28695, GGB28866, and GGB45624 (q = 0.004222573, q = 0.02492251, and q = 2.899739 × 10^−5^, respectively).

At the species level, 24 out of 89 taxa were significantly differentially abundant in ANCOM-BC2 analyses. Three species were more abundant in the mice on supplemented diets regardless of sex, including two undetermined species of Clostridia in the *Oscillospiraceae* and one entirely unclassified bacterial species (SGB102200 q = 2.003275 × 10^−24^, SGB43546 q = 6.522887 × 10^−7^, and SGB41664 q = 1.355927 × 10^−5^, respectively). Three more species were also more abundant in the mice fed with ammoniated diets and also statistically more abundant in the males, including the *Peptostreptococcaceae* species *Romboutsia ilealis*, and two Firmicutes species of undetermined taxonomic class (SGB43516 and SGB63337) (diet: q = 0.001698942, q = 3.008686 × 10^−4^, and q = 1.376109 × 10^−10^, respectively; sex: q = 0.03078539, q = 0.03527578, q = 5.878598 × 10^−19^, respectively).

Six species were significantly more abundant in the control mice. Three of these were more abundant in the control mice regardless of sex, including the Firmicutes taxa *Lactococcus cremoris*, an *Oscillospiraceae* species (SGB42542), and an unclassified bacterium named ‘bacterium_1XD42_54’ (q = 6.711283 × 10^−9^, q = 3.481441 × 10^−4^, q = 8.920275 × 10^−4^, respectively). The Betaproteobacteria species SGB44464 in the family *Rhodocyclaceae* was more abundant in the controls and in the females (diet: q = 1.234079 × 10^−4^, sex: q = 0.0149627), and two Firmicutes species (SGB63186 and SGB41518) were more abundant in the controls and males (diet: q = 0.03688538, q = 1.432517 × 10^−9^; sex: q = 0.04379997, q = 0.04651438, respectively).

Other species were differentially abundant between the sexes, regardless of treatment. For example, the females had significantly more of two species of Bacteroidetes in the family *Muribaculaceae* (SGB40338 and SGB63927) and a species of Firmicutes in the family *Peptostreptococcaceae* (SGB42492) (q = 3.556715 × 10^−3^, q = 5.71868 × 10^−8^, q = 2.007916 × 10^−6^, respectively). Similarly, eight species, all Firmicutes, were significantly more abundant in the males, regardless of treatment: five species of undetermined taxonomic class (SGB41712, SGB41703, SGB41304, SGB41326, and SGB41538) (q = 3.285611 × 10^−3^, q = 0.0149627, q = 0.03527578, q = 8.956676 × 10^−4^, and q = 4.315426 × 10^−3^, respectively), an *Oscillospiraceae* SGB43537 (q = 0.0149627), a *Clostridiaceae* SGB43072 (q = 0.02012161), and a species designated *Anaerotruncus* sp. 1XD42_93 (q = 9.835964 × 10^−51^).

### 3.8. Metapangenomics and Gene Ontology (GO) Enrichment Analysis

**Figure 9:**
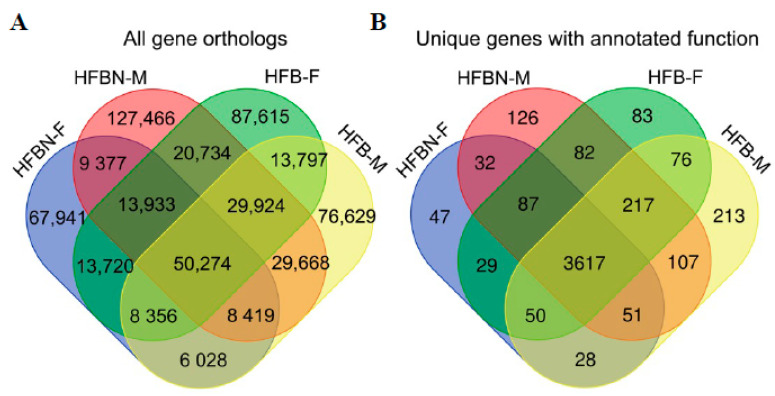
Metapangenomes for microbiomes from mice fed on high-fat beef diets with ammoniation (HFBN-F, females; HFBN-M, males) or unsupplemented diets (HFB-F, females; HFB-M, males). (**A**) All orthologous clusters; (**B**) orthologous clusters with unique functional annotation remaining after removal of duplicated or variant gene versions and genes of unknown function (i.e., ‘hypothetical proteins). Numbers indicate distinct ortholog clusters from Roary.

Ortholog analysis for all annotated genes, including genes with unknown function, produced 563,881 gene clusters (Figure 9A, Supplementary Table S3). The majority of these gene clusters (99.14%) were variant copies (e.g., homologs of the same function in divergent microbes) or genes with unknown function. Gene clusters with unknown function (i.e., annotated as ‘hypothetical protein’) comprised 78.21% of total gene clusters. Removing genes with unknown function and consolidating variant copies prior to gene ontology enrichment analysis resulted in only 4845 gene clusters that had unique and known functional annotation (Figure 9B). For the total orthologous cluster and reduced cluster lists, pangenome overlap was analyzed and depicted in Venn diagrams, revealing 50,274 gene clusters (8.916%) (Figure 9A) and 3617 gene clusters (74.65%) (Figure 9B) that were ‘core genes’ (i.e., universally shared) across groups for the list of all orthologs and the uniquely annotated genes, respectively. For the metapangenomic analysis including all gene clusters (Figure 9A), there were large numbers of unshared ‘cloud’ genes unique to only one group—for example, 359,651 genes (63.78%) for the set of all gene orthologs (Figure 9A), and 527 genes (10.88%) for the reduced set of orthologs (Figure 9B).

The number of shared gene clusters unique to the ammonium-supplemented diet treatments was lower than that shared uniquely among the controls for the comparison with all genes (9,377 shared genes in HFBN versus 13,797 shared genes in HFB) and for genes with uniquely annotated function (32 shared genes in HFBN versus 76 shared genes in HFB) (Figure 9). In both pangenome comparisons, unshared ‘cloud’ genes were higher than the numbers of clusters shared uniquely among the treatment groups (Figure 9). A similar pattern was observed when data were grouped by sex: the number of shared genes was lower in the females than in the males for the comparison with all genes (13,720 shared genes in the females versus 29,668 shared genes in the males) and for unique genes with annotated function (29 shared genes in the females versus 107 shared genes in the males). In both pangenome comparisons, unshared ‘cloud’ genes were higher than the numbers shared uniquely among the treatment groups (Figure 7).

**Figure 10:**
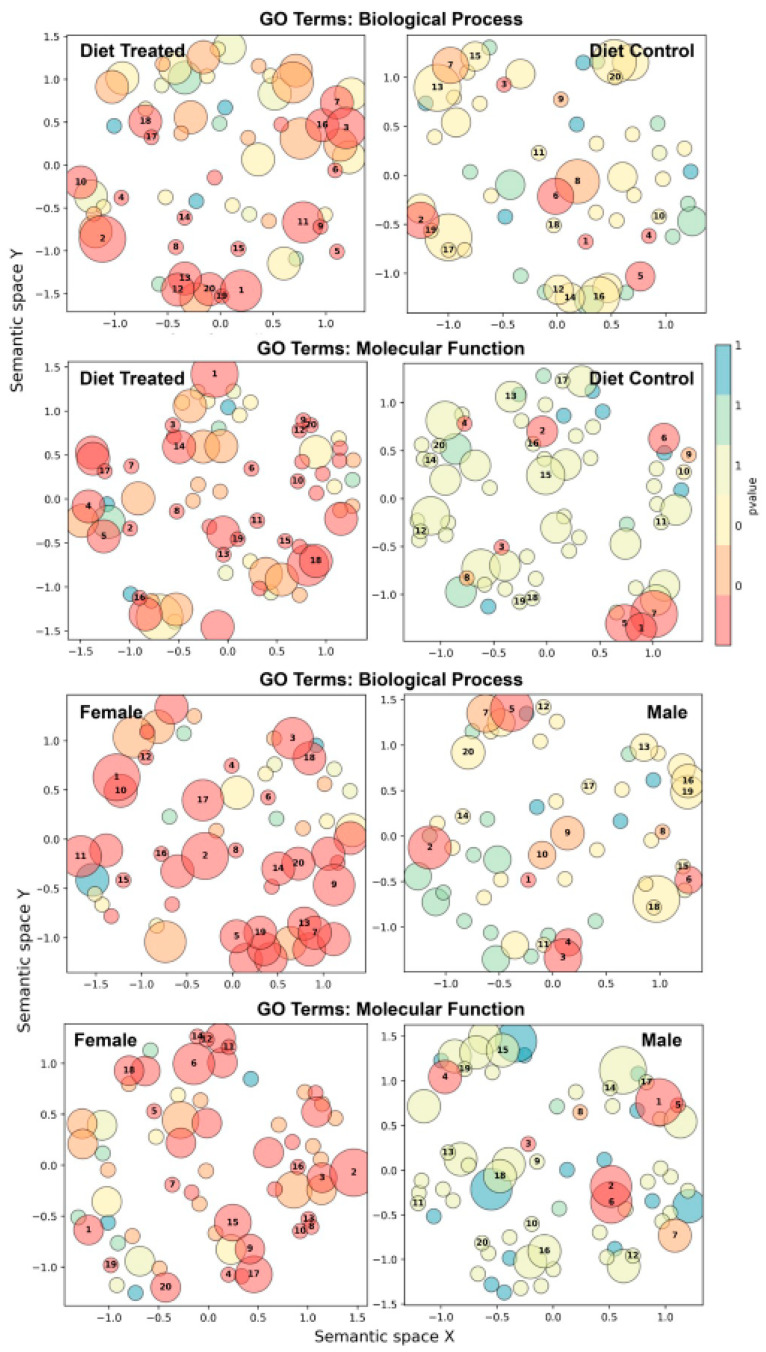
Patterns of significantly enriched gene ontology (GO) terms, depicted in semantic space using ‘GO-figure!’ for mice fed on ammoniated beef protein, high-fat beef diets (HFBN-F, females; HFBN-M, males) or unsupplemented diets (HFB-F, females; HFB-M, males). Circle size represents the relative number of genes per GO term, and numbers in circles represent the terms with lowest p-values (see Supplementary Table S3).

Gene ontology (GO) enrichment analyses comparing the ‘cloud’ and uniquely shared genes among enhanced pH diet groups (HFBN) compared to the controls (HFB) revealed differences in GO enrichment (Table 4, Supplementary Table S2) and highly divergent GO term semantic space patterns (Figure 10, Supplementary Table S3). The ammonium-supplemented diet mice had microbiomes that were enriched for GO biological processes, such as glycine betaine transport, xenobiotic detoxification by transmembrane export across the plasma membrane, defense response to bacteria, nitrate assimilation, and anaerobic respiration (Table 4). They were also enriched for GO molecular functions, such as phosphorelay sensor kinase activity, phosphopantetheine binding, lipoic acid binding, catalytic activity, toxin activity, and protein serine/threonine kinase activity. Additional GO terms enhanced in pH diet groups compared to the control diets in GO functions (semantic space) include positive regulation of cell proliferation, and L-fucose, alkane, and homocysteine catabolic processes, among others (Figure 10).

Unsupplemented diet (HFB), compared to ammoniated protein diet (HFBN), microbiomes were significantly enriched for GO biological processes such as glutathione metabolic process, response to oxidative stress, ion transport, respiratory electron transport chain, organic phosphonate metabolic process, polysaccharide transport, sucrose metabolic process, and aromatic compound catabolic process (Table 4). They were enriched for GO molecular functions, such as transmembrane transporter activity, glutathione transferase activity, porin activity, copper ion binding, carbohydrate:proton symporter activity, glucose transmembrane transporter activity, and flavin adenine dinucleotide binding. Additional GO semantic space differences that were enhanced in microbiomes from the control diets include toxin transport, fatty acid elongation, polysaccharide transport, sucrose metabolic process, and so on (Figure 10).

The females, compared to the males, were enriched for GO biological processes, such as response to toxic substance, aerobic electron transport chain, nitrate assimilation, carotenoid biosynthetic process, and heme metabolic process (Table 4). The females had microbiomes enhanced for GO molecular functions, such as FAD binding, fatty acid binding, and N-acetylmuramoyl-L-alanine amidase activity. Additional GO semantic space differences that were enhanced in microbiomes from the females include the S-methylmethionine cycle, 2,34,6-trinitrotoluene catabolic process, glycolate catabolic process, inositol biosynthetic process, mercury ion binding, cytochrome-c oxidase activity, and so on (Figure 10).

The males were different from females in GO biological process enrichment for glutathione metabolic process, glycine betaine transport, response to oxidative stress, organic phosphonate metabolic process, polysaccharide transport, and so on (Table 4), while enriched molecular functions included phosphopantetheine binding, L-phosphoserine phosphatase activity, porin activity, flavin adenine dinucleotide binding, 3-oxoacyl-[acyl-carrier-protein] synthase activity, and so on. Additional GO semantic space enrichment in males included the cellulose biosynthetic process, beta-ketoadipate pathway, organic phosphonate catabolic process, and so on (Figure 10).

## 4. Discussion

The main goal of this study was to test the effects of dietary beef protein ammoniation —prior to its addition into the complete diet—on microbiome composition and function in mice fed on high-fat diets in order to expand on data and theory that suggest the benefits of ammoniated diets interacting through microbiome changes [2,4,5,16,17]. This is an important point to consider. Modifying the protein before folding it into the dietary mix showed profound changes to the microbiota of the mice. Following mouse survivability trends during the preceding 12 months, we decided that it was prudent to initiate microbiome assessment at 16 months, 2 months earlier than initially planned, as sampling cohorts for HFB-M after 12 months were declining rapidly. This choice was affirmed as HFB-F and HFBN-F also began to decline after16 months.

We considered food consumption over time, total progressive weight gain, and the ratios of lean to fat mass over time. We also included Kaplan–Meier survivorship curves. While the main data analysis was over microbiome data, with respect to food consumption, the males maintained the largest mass regardless of diet until around week 28. Notably, the females had much lower total body mass than the males for the duration of the study. We included references to manuscripts in preparation for publication that include extensive additional phenotype data associated with weight gain, survivorship, cancer incidence, and changes in adipokine secretions correlated with diet (Gollahon—unpublished data, manuscripts in preparation). When assessing the trends in total mass change over time, it is apparent from the fluctuations in HFB-M that the mice began losing weight as their health declined until they were removed from the study. These occurrences are less apparent in HFBN-F and HFB-F, but are still present. We hypothesized that ammonium supplementation of beef protein in high-fat beef diets would reduce dietary acid load and shift microbiome community diversity and function. Our results showed statistically significant changes in microbiome taxa and microbiome functions depending on diet, with several health-associated microbiome changes in the ammonium-supplemented diets. Consistent with previous studies [51,52,53], we also found that sex was a confounding factor that also affected microbiome responses to ammonium-supplemented high-fat beef diets, and this was further supported by the difference in total mass accumulation and survivability.

Despite overlap in many taxa among treatments, we found significant differences in microbiomes at the phylum, genus, and species level associated with ammonium-supplemented high-fat beef diets, which may have important implications. For example, we found that the phylum Actinobacteria was less abundant in ammonium supplemented diets than in the controls. This result suggests that supplementation attenuated the negative effects of high fat, given previous studies showing higher Actinobacteria to be a biomarker of high-fat diets [54]. Even though Actinobacteria (particularly *Bifidobacteria*) are typically proportionally less abundant than other taxa, they generally have significant effects on microbiome function and disease [55]. Our results also support previous findings suggesting that the dominant actinobacterial family *Eggerthellaceae* and its dominant genus *Adlercreutzia* are associated with increased BMI and obesity [56]. High-fat diet-associated co-morbidities [57,58,59,60]. Another notable finding was the abundance of the species *Pediococcus acidilactici* (*Lactobacillaceae*) in the ammonium-supplemented diet males. This species has been used for its probiotic properties, specifically its ability to secrete bacteriocin and lactic acid, which can limit pathogen growth and promote immunity in the gut [61], and its abundance in these males may explain the relatively lower abundance of Verrucomicrobiota, as the antimicrobial properties of *A. muciniphila* and *P. acidilactici* are similar [62].

A notable betaproteobacterium that differed between diet groups was a genus of *Rhodocyclaceae*. This taxon was previously shown to increase in high-fat diet mice when treated with alpha linoleic acid, and its abundance was positively correlated with the expression of the gut mucosal tight junction protein zona occludens-1 [63]. The implications of its higher abundance in the controls (and females) in the current study are unclear.

Although the diet-associated differences above were significant, regardless of sex, our data showed that sex had a significant role in the microbiome community profile. Indeed, significant sex differences in metabolic outcomes were found in a recent study on these diets, although the diet was beneficial overall for both males and females [5]. In the current study, we found that some species differed between sexes, regardless of treatment, including members of the Bacteroidetes family *Muribaculaceae* (previously known as S24–S27), which was more abundant in the female mice in our study. This taxon has been extensively studied as a group of mucus metabolizers, which typically make up some of the most abundant species of the murine gut [64]. Our results are consistent with previous studies showing that these important mucosa-associated microbes occur at higher levels in females [53] likely because of female hormones. In combination with F/B ratio differences, studies have suggested that *Muribaculaceae*, as highly efficient carbohydrate degraders, help females regulate energy balance more rapidly [53] and some species not yet described can be protective against high-fat diet-induced obesity when there is sufficient dietary oligofructose [65].

We found that several species (mostly undetermined species of Firmicutes) were more abundant in the males, regardless of diet. However, of particular interest was the species *Anaerotruncus*. Previous studies have shown that this succinate-producing microbe, which has also been isolated from humans in numerous studies, is found at higher levels in male mice [66,67] and responds negatively to estrogen. The males also had statistically lower levels of ‘Other phyla’ (rare phyla) compared to the females, which may reflect differences in higher-level taxonomic diversity that is sex-dependent, and possibly related to changes observed in other studies related to aging mice [68]. Together, our findings of sex differences in these microbiomes were not surprising, given previous studies [51,52,53].

Although differences in *Akkermansia* (Verrucomicrobiota) were not statistically significant among our diet treatments, it was remarkable to find this well-studied beneficial mucin-degrading and obesity-related disease-reducing species at such high abundance in all of our mice. *Akkermansia muciniphilia* strains and diverse closely related species, which typically represent between 1 and 4% of the bacteria in human and mouse feces, have been extensively shown to increase in abundance with healthy diets [69,70,71,72,73]. However, *Akkermansia* was found at 56–86% in our Centrifuge-binned metagenomic data (and 30–69% in our MetaPhlAn4-classified metagenomic data), suggesting unexpected and unprecedented dominance of this taxon, not previously observed except in unusual cases such as following treatment with broad-spectrum antibiotics [74] and a single study on 18-month-old mice fed on chow diets [68]. Although, like the latter study, our mice were sampled at an older age (16 months), most studies of *Akkermansia* suggest that this bacteria tends to decrease, rather than increase, with age and is lower in mice fed on high-fat diets [75]. However, a few studies suggest the microbe can increase up to 3-fold with healthy aging [76,77]. Still, the rate of *Akkermansia* in older mice is typically not more than ~10% on healthy diets [78]. However, at least one study showed that under fecal transplantation conditions, *Akkermansia* can dramatically rise with age [79] and reduce senescence. Perhaps most relevant to our findings is a study showing *Akkermansia* levels in mice fed beef protein diets averaging 12.68%, but with ranges of up to 52% in some individuals, suggesting a potentially significant effect of protein type [80]. However, this high rate of *Akkermansia* was seen in lean-fat beef-fed mice, whereas high-fat beef diet mice had more modest and variable levels of this bacteria. Another potential factor that could explain our findings is the mouse strains used, given that studies suggesting *Akkermansia* rates vary between mouse strains [23,51]. Taken together, in the context of what others have found, we suggest that our results show potentially important and not previously reported interactions between aging, beef protein diets, and high-fat diets, which may lead to a rise in this beneficial microbe. Moreover, the most dramatic rise in *Akkermansia* was found in the females on ammonium-supplemented high-fat beef diets, warranting more research on this potentially beneficial result.

Even with the small number of groups in this study, we found significant metapangenomic patterns with 4845 annotated gene clusters that could be analyzed for GO functional enrichment. Interestingly, the number of shared gene clusters unique to the ammonium-supplemented diet microbiomes was lower than that in the unsupplemented high-fat beef diets. This was consistent with greater functional dysbiosis on the unsupplemented high-fat diets compared to the treatments—consistent with the so-called Anna Karenina principle [81,82], which is the observation that dysbiosis has more diverse manifestations than does a healthy microbiome, and implying that ammonium supplementation of these diets may have reduced gut dysbiosis.

Among the top functions enriched in ammonium-supplemented high-fat beef diet-treated mice microbiomes were glycine betaine transport, xenobiotic detoxification, and defense response to bacterium—functions that have been shown to be protective, with positive health consequences. For example, glycine betaine is osmoprotective against dehydration [83] and its conversion to betainized compounds is an important microbiome process affected by dietary fiber [84], while genes in glycine betaine transport may contribute to trimethylamine homeostasis, which benefits cardiovascular health [85]. Xenobiotic detoxification functions have been shown to be enhanced in the microbiomes of long-lived individuals [86], suggesting a positive role. Enhanced defense responses in our treatment microbiomes are consistent with studies of gene expression, suggesting the important role of defense supporting immune responses to the negative impacts of high-fat diets [10].

Further enriched functions in the ammonium-supplemented group included homocysteine catabolism, nitrate assimilation, anaerobic respiration, lipoic acid binding, and protein serine/threonine kinase activity—functions that have been reported as being associated with major microbiome benefits in past studies. For example, increased homocysteine catabolism may be a positive response to increased homocysteine, which has been found to be correlated with conditions contributing to obesity [87]. Nitrate assimilation may be a sign of enhanced response to nitrates, which have been a focus for the development of beneficial prebiotics that alter the oral microbiome [88]. Anaerobic respiration, which was enhanced in ammoniated protein diets compared to unammoniated diets in our study, could be a biomarker of improved host control of anaerobic fermentation, as part of maintaining healthy microbiome redox homeostasis [89]. Enhanced lipoic acid binding could indicate health-promoting changes given that lipoic acid binding sites are major epitopes for immune cells [90]. Finally, enhancement of protein serine/threonine kinase activity may reflect improvement in levels of the beneficial and protective SCFA butyrate, which has been shown to be a regulator of this GO activity [91].

Functions that were notably enriched in the unsupplemented high-fat beef diet group included glutathione metabolic process and glutathione transfer activity, response to oxidative stress, and several polysaccharide/sucrose metabolic activities—together indicative of several less healthy or obesity-associated functions. For example, a recent study showed that glutathione metabolic processes were enhanced in obese compared to lean children’s microbiomes [92]. Oxidative stress is a major factor in high-fat diets, with studies showing microbiome shifts in response to this factor [93]. Others have shown that oxidative stress microbiome markers are a major factor in Western diet [94]. The higher abundance of Bacteroidetes in the high-fat beef diet group may help explain enrichment for polysaccharide transport, sucrose metabolic process, carbohydrate:proton symporter activity, and glucose transmembrane transporter activity, as a major part of Bacteroidetes (especially *Bacteroides* spp.) genomes are polysaccharide and sugar metabolism genes [95], inversely associated with metabolic diseases.

In the unsupplemented high-fat beef diet group, we also found that microbiomes were enriched for porin activity, toxin transport, ion transport, and organic phosphonate metabolism—functions that may suggest adverse gut health. For example, porin activity and toxin transport tend to be associated with increased pathogens or microbial virulence, as seen in a study on advanced colorectal cancer [96]. Enhanced ion transport and respiratory electron transport chain activity in the microbiome is an unexpected finding. However, typically, disruption of host ion transport and host respiratory electron transport chain function are responses to gut dysbiosis. It may be possible that metabolic conditions or signals in dysbiosis also shift the microbiome in the same way. Finally, the observation of enhanced organic phosphonate metabolic process is of interest, as this is typical in metagenomes disrupted by organic phosphonate components of agrochemicals (e.g., pesticides and flame retardants) [97].

Altogether, this study uncovered patterns in microbiome change associated with ammonium-supplemented diets; however, the study had several limitations. As with most microbiome studies [41,42] we found that a large portion of the taxa and genes were unique to this study and, notably, 78.21% of predicted genes could not be mapped to references in available databases, which limited our ability to interpret some of these data. While using shotgun metagenomic data was an improvement over amplicon sequencing approaches in that it was more sensitive and could recover strain-level data and gene functional data, the added cost per sample limited the sample size that was feasible in this project, reducing the power of our statistical tests. Thus, our study should be interpreted as preliminary and can be used as a basis to design future studies, which, given our results showing differences in response to ammonium-supplemented dietary beef protein that depend on sex, should pay special attention to obtaining sufficient sample sizes from both males and females. Finally, while we uncovered differences in inferred functional potential from different gene repertoires, future studies will be needed to confirm active metabolic differences using methods such as metatranscriptomics or metabolomics.

## 5. Conclusions

In summary, this study was novel in that it examined the relationship between the gut microbiome and an ammonium-supplemented diet by modifying the beef protein component. Together, the results suggested that in aging mice fed a high-fat beef diet, the beneficial mucin degrader *Akkermansia muciniphila* was dominant, especially in ammonium-supplemented diet females, and that numerous other taxa, including *Romboutsia*, *Oscillospiraceae*, and *Lactococcus cremoris*, known to be associated with health benefits, were more abundant in ammonium-supplemented diet microbiomes. Similarly, we identified a range of putatively beneficial microbiome functions associated with ammonium supplementation, such as glycine betaine transport, xenobiotic detoxification, enhanced defense, and others. In contrast, high-fat beef diets were enriched for a set of disease-associated microbiome functions, including those associated with obesity and metabolic diseases. These outcomes warrant further study of ammonium-supplemented proteins as candidates for improving health by shifting the microbiome.

## Figures and Tables

**Table 1:** Compositions of the HFBN and HFB diets.

Ingredient	HFBN Dietary Components	HFB Dietary Components
Ammonium-supplemented beef (cooked, freeze-dried)	352.42 g	0 g
Unsupplemented beef (cooked, freeze-dried)	0 g	293.64 g
L-cystine	3 g	3 g
Corn starch	68.68 g	68.46 g
Maltodextrin 10	100 g	100 g
Sucrose	175.21 g	175.21 g
Cellulose	45.07 g	48.53 g
Soybean oil	25 g	25 g
Beef fat, Bunge	23.28 g	78.76 g
Mineral mix S10026A (No Ca, P, K, Na, Cl)	5 g	5 g
Dicalcium phosphate	13 g	13 g
Calcium carbonate	5.5 g	5.5 g
Potassium citrate, 1 H_2_O	16.5 g	16.5 g
Sodium chloride	1.088 g	0.87 g
Vitamin mix V10001	10 g	10 g
Choline bitartrate	2 g	2 g
Cholesterol	0 g	0.02 g
Yellow dye #5 FD&C	0 g	0.025 g
Red dye #40 FD&C	0.025 g	0.025 g
Blue dye #1 FD&C	0.025 g	0 g
TOTAL MASS	845.798 g	845.54 g
Protein	18% of Kcal	18% of Kcal
Carbohydrates	36% of Kcal	36% of Kcal
Fats	46% of Kcal	46% of Kcal
TOTAL Kcal%	100% of Kcal	100% of Kcal

**Table 2:** Average food consumed in grams per mouse per week for the study duration. Standard error separated by group.

	Average Food Consumed per Mouse (g)	Standard Error
HFBN-F	15.3	±0.02
HFBN-M	21.4	±0.04
HFB-F	16.8	±0.03
HFB-M	19.8	±0.03

**Table 3:** Shotgun metagenomic sequencing data and assembly and annotation details for fecal microbiomes from mice fed high-fat beef diets with ammonium supplementation (HFBN-F, females; HFBN-M, males) or unsupplemented diets (HFB-F, females; HFB-M, males).

	Raw Reads (Millions)	Number of Contigs	Maximum Contig Length	Total Contig Length (Mbp)	Assembly N50	Number of Genes Annotated	Unique Gene Orthologs with Known Function
HFBN-F	76.80	180,626	457,219	173.05	2355	176,033	3931
HFBN-M	66.90	342,839	447,818	322.73	2837	325,674	4319
HFB-F	62.73	219,182	684,229	246.30	2575	213,531	4214
HFB-M	62.32	250,995	447,727	257.89	3141	239,786	4359

**Table 4:** Significantly enriched gene ontology (GO) terms for metapangenomes of fecal microbiomes from the mice. Comparison 1—mice fed HFB ammoniated diets compared to unsupplemented diets (combining males and females). Comparison 2—females compared to males (combining the two diets).

Ammonium Beef Diet (HFBN)	*p*-Value	Control Diet (HFB)	*p*-Value
Comparison 1			
GO Biological Process:		GO Biological Process:	
glycine betaine transport	0.0042	glutathione metabolic process	0.0041
xenobiotic detoxification by transmembrane export across the plasma membrane	0.0092	response to oxidative stress	0.0075
defense response to bacterium	0.0198	ion transport	0.0185
nitrate assimilation	0.0371	respiratory electron transport chain	0.0331
anaerobic respiration	0.0371	organic phosphonate metabolic process	0.0451
		polysaccharide transport	0.0457
		sucrose metabolic process	0.0457
		dormancy process	0.0457
		aromatic compound catabolic process	0.0472
GO Molecular Function:		GO Molecular Function:	
phosphorelay sensor kinase activity	0.00068	transmembrane transporter activity	0.0023
phosphopantetheine binding	0.00882	glutathione transferase activity	0.0036
lipoic acid binding	0.01292	porin activity	0.0066
catalytic activity	0.02998	copper ion binding	0.0162
toxin activity	0.03361	carbohydrate:proton symporter activity	0.024
protein serine/threonine kinase activity	0.03361	glucose transmembrane transporter activity	0.0469
		flavin adenine dinucleotide binding	0.0487
GO Cellular Component:		GO Cellular Component:	
integral component of membrane	0.019	pore complex	0.00089
		cell outer membrane	0.00107
		periplasmic space	0.00349
		ATP-binding cassette (ABC) transporter complex	0.01176
		integral component of membrane	0.0157
**Comparison 2**			
**Females**	***p*-Value**	**Males**	***p*-Value**
GO Biological Process:		GO Biological Process:	
response to toxic substance	0.012	glutathione metabolic process	0.004
aerobic electron transport chain	0.013	ion transport	0.018
nitrate assimilation	0.023	glycine betaine transport	0.031
carotenoid biosynthetic process	0.024	response to oxidative stress	0.036
heme metabolic process	0.031	organic phosphonate metabolic process	0.044
		polysaccharide transport	0.045
		dormancy process	0.045
		cell adhesion	0.049
GO Molecular Function:		GO Molecular Function:	
FAD binding	0.032	phosphopantetheine binding	0.0011
fatty acid binding	0.032	glutathione transferase activity	0.0035
*N*-acetylmuramoyl-l-alanine amidase activity	0.039	l-phosphoserine phosphatase activity	0.0035
		porin activity	0.0065
		flavin adenine dinucleotide binding	0.0168
		3-oxoacyl-[acyl-carrier-protein] synthase activity	0.0236
		phosphorelay sensor kinase activity	0.0375
		anti-sigma factor antagonist activity	0.0463
		GO Cellular Component:	
		pore complex	0.00091
		cell outer membrane	0.00425
		periplasmic space	0.01042
		integral component of membrane	0.0165
		outer membrane	0.04496

## Data Availability

All sequence data from fecal samples have been deposited in the Sequence Read Archive (SRA) under BioProject number PRJNA1085270. All scripts used for data analysis are available in Supplementary File S1. Raw data and R code for the Kaplan–Meier curve are available at: https://github.com/BenjaminBarr/HFB-HFBN/releases/tag/v1.1 (accessed on 15 March 2024).

## Supplementary Materials

The following supporting information can be downloaded at: https://www.mdpi.com/article/10.3390/nu16111613/s1, File S1: Computer code used in this study; Figure S1: Relative abundances of microbiome phyla after taxonomic binning and filtering for nearly complete metagenomes for mice fed on high fat beef diets with pH-enhancement (HFBN-F, females; HFBN-M, males) or without pH-enhancement (HFB-F, females; HFB-M, males). Only confidently assigned taxa are included; Figure S2: Abundance-GC plots created from metaWRAP’s Blobology module for mice fed on high fat beef diets with pH-enhancement (HFBN-F, females; HFBN-M, males) or without pH-enhancement (HFB-F, females; HFB-M, males). Colors assigned based on phylum association; Table S1: Complete orthologous gene results from Roary analysis; Table S2: Significantly enriched gene ontology (GO) categories from TopGO analysis for comparisons between supplemented (HFBN) and unsupplemented (HFB) high-fat beef diet microbiomes and males and females; Table S3: Gene ontology (GO) categories plotted in GO-Figure for numbered categories shown in circles in Figure 8.
