# Supplementary material for: Microbiome Taxonomic and Functional Differences in C3H/HeJ Mice Fed a Long-Term High-Fat Diet with Beef Protein ± Ammonium Hydroxide Supplementation
Source: Nutrients. 2024 May 25;16(11):1613. doi: 10.3390/nu16111613 (PMC11174526; doi:10.3390/nu16111613)
Supplement: Supplementary file 1 [file nutrients-16-01613-s001.zip › Supplementary Figure S1.pdf]

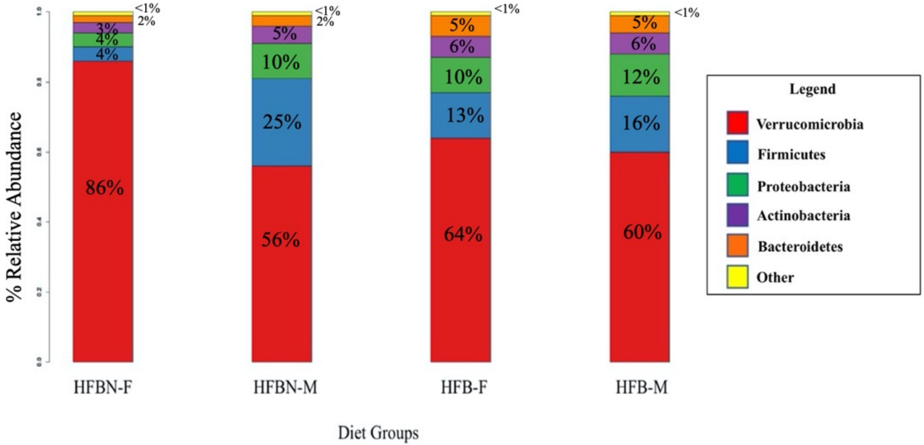

Supplementary Figure S1: Relative abundances of microbiome phyla after taxonomic binning and filtering for nearly complete metagenomes for mice fed on high fat beef diets with pH-enhancement (HFBN-F, females; HFBN-M, males) or without pH-enhancement (HFB-F, females; HFB-M, males). Only confidently assigned taxa are included.
