# Supplementary material for: Microbiome Taxonomic and Functional Differences in C3H/HeJ Mice Fed a Long-Term High-Fat Diet with Beef Protein ± Ammonium Hydroxide Supplementation
Source: Nutrients. 2024 May 25;16(11):1613. doi: 10.3390/nu16111613 (PMC11174526; doi:10.3390/nu16111613)
Supplement: Supplementary file 1 [file nutrients-16-01613-s001.zip › Supplementary File S1_Code.pdf]

Supplementary File S1: Computer code used in this study.

READ FILTERING AND ASSEMBLY:

Read merging with PEAR:

```
path=/media/DataA/Emily/all_samples/

for i in ${path}/*_001.fastq
do
   name=$(basename ${i%_R1_001.fastq})

   pear -j 46 -f ${path}/${name}_R1_001.fastq -r ${path}/${name}_R2_001.fastq -o ${name} >
${name}.stats
done
```

Read trimming with Trimmomatic:

```
INDIR=/media/DataA/Emily/all_samples

for i in ${INDIR}/*.assembled.fastq
do
   name=$(basename ${i%.assembled.fastq})
   java -jar /opt/Trimmomatic-0.38/trimmomatic-0.38.jar SE -threads 46 -phred33
${INDIR}/${name}.assembled.fastq ${name}_SE.fastq ILLUMINACLIP:/opt/Trimmomatic-
0.38/adapters/TruSeq3-SE.fa:2:30:10:2:keepBothReads LEADING:20 TRAILING:20
SLIDINGWINDOW:4:20 MINLEN:80 1> ${name}_SE.out 2> ${name}_SE.trimstats
   java -jar /opt/Trimmomatic-0.38/trimmomatic-0.38.jar PE -threads 46 -phred33
${INDIR}/${name}.unassembled.forward.fastq ${INDIR}/${name}.unassembled.reverse.fastq
${name}_R1p.fastq ${name}_R1up.fastq ${name}_R2p.fastq ${name}_R2up.fastq
ILLUMINACLIP:/opt/Trimmomatic-0.38/adapters/TruSeq3-PE.fa:2:30:10:2:keepBothReads LEADING:20
TRAILING:20 SLIDINGWINDOW:4:20 MINLEN:80 1> ${name}_PE.out 2> ${name}_PE.trimstats
   chmod +x ${name}_SE.trimstats ${name}_PE.trimstats ${name}_SE.out ${name}_PE.out
done
```

De novo assembly with MetaSPAdes:

```
/opt/SPAdes-3.13.0-Linux/bin/metaspades.py -t 46 -m 370 -k 21,33,45,59,73,99,111 --disable-gzip-output
--pe1-m /media/DataA/Emily/Group4Female.singleTRIM.fastq --pe1-1
/media/DataA/Emily/Group4Female_R1pTRIM.fastq --pe1-2
/media/DataA/Emily/Group4Female_R2pTRIM.fastq -o
/media/DataA/Emily/Assembled_Data/Assembly_Group4Female
```

TAXONOMIC BINNING WITH METAWRAP:

Initial binning with metaWRAP:

```
ASSEMBLY_DIR="/media/DataA/Emily/Assembled_Data/Scaffolds"
READ_DIR="/media/DataA/Emily/all_samples"
```

```
OUTDIR="/media/DataA/Emily/Initial_Binning"

# source activate metawrap-env

for x in ${ASSEMBLY_DIR}/*Scaffold.fasta
do
name=$(basename ${x%Scaffold.fasta})
metawrap binning -o ${name} -t 46 -m 300 -a ${ASSEMBLY_DIR}/${name}Scaffold.fasta --metabat2 --
maxbin2 --concoct ${READ_DIR}/${name}_1.fastq ${READ_DIR}/${name}_2.fastq --single-end
${READ_DIR}/${name}.singleTRIM.fastq* 1>  ${name}.log 2> ${name}.err
done
```

Bin refinement with metaWRAP:

```
INDIR=/media/DataA/Emily/Initial_Binning

# source activate metawrap-env

for i in ${INDIR}/*.log
do
   name=$(basename ${i%.log})
   metawrap bin_refinement -o ${name} -t 46 -m 360 -A ${INDIR}/${name}/metabat2_bins -B
${INDIR}/${name}/maxbin2_bins -C ${INDIR}/${name}/concoct_bins -c 90 -x 10 1> ${name}.log 2>
${name}.err
done
```

Bin reassembly with metaWRAP:

```
BINS=/media/DataA/Emily/BinRefine
ASSEMBLY=/media/DataA/Emily/Assembled_Data
READS=/media/DataA/Emily/PTReads/

# source activate metawrap-env

for i in ${BINS}/*.log
do
   name=$(basename ${i%.log})
   metawrap reassemble_bins -t 46 -m 360 -c 90 -x 10 -o ${name} -b
${BINS}/${name}/metawrap_90_10_bins -1 ${READS}/${name}_1.fastq -2 ${READS}/${name}_2.fastq 1>
${name}.log 2> ${name}.err
done
```

Quant bin with metaWRAP:

```
# source activate metawrap-env

for i in /media/DataA/Emily/BinRefine/*.log
do
   name=$(basename ${i%.log})
```

```
    metawrap quant_bins -b /media/DataA/Emily/BinRefine/${name}/metawrap_90_10_bins -o
${name}_QUANT_BINS -a /media/DataA/Emily/Assembled_Data/Scaffolds/${name}Scaffold.fasta
/media/DataA/Emily/PTReads/${name}_1.fastq /media/DataA/Emily/PTReads/${name}_2.fastq

done
```

Bin classify with metaWRAP:

used these outputs to double check the Centrifuge results

```
BINS=/media/DataA/Emily/Bin_Reassembly

# source activate metawrap-env

for i in ${BINS}/*.log
do
    name=$(basename ${i%.log})
    metawrap classify_bins -t 46 -o ${name} -b ${BINS}/${name}/reassembled_bins 1> ${name}.log 2>
${name}.err
done
```

TAXONOMIC BINNING WITH CENTRIFUGE AND KRONA:

Centrifuge:

instructions here: https://genomics.sschmeier.com/ngs-taxonomic-investigation/index.html
##Ran as a 1-liner

```
#conda activate centrifuge

$ centrifuge -x p_compressed+h+v -1 /home/mclewis/emily/PTReads/Group3Female_1.fastq  -2
/home/mclewis/emily/PTReads/Group3Female_2.fastq --report-file G3F-evol1-report.txt -S G3F-evol1-
results.txt
```

Visualizing Centrifuge w/ Krona:

same link as above for Krona installation instructions
##Ran this as a 1-liner

```
#conda activate krona

$ cd centrifuge
$ cat G3F-evol1-results.txt | cut -f 1,3 > G3F-evol1-results.krona
$ ktImportTaxonomy G3F-evol1-results.krona
$ firefox taxonomy.krona.html

#Need to scp the .html files to local computer then open the web link
```

TAXONOMIC BINNING WITH METAPHLAN4:

#install and activate

conda activate mpa

launching metaphlan

```
metaphlan ../all_samples/Group3Male_R2_001.fastq,../all_samples/Group3Males.unassembled.forward.fastq,../all_samples/Group3Males.assembled.fastq --bowtie2out Male3.bowtie2.bz2 --nproc 48 --input_type fastq -o profiled_Male3.txt

metaphlan ../all_samples/Group3Female.assembled.fastq.gz,../all_samples/Group3Female.unassembled.forward.fastq.gz,../all_samples/Group3Female.unassembled.reverse.fastq.gz --bowtie2out Female3pear.bowtie2.bz2 --nproc 48 --input_type fastq -o pear_Female3.txt

metaphlan ../all_samples/Group4Female.assembled.fastq.gz,../all_samples/Group4Female.unassembled.forward.fastq.gz,../all_samples/Group4Female.unassembled.reverse.fastq.gz --bowtie2out Female4pear.bowtie2.bz2 --nproc 48 --input_type fastq -o pear_Female4.txt

metaphlan ../all_samples/Group3Males.assembled.fastq,../all_samples/Group3Males.unassembled.forward.fastq,../all_samples/Group3Males.unassembled.reverse.fastq.gz --bowtie2out Male3pear.bowtie2.bz2 --nproc 48 --input_type fastq -o pear_Male3.txt

metaphlan ../all_samples/Group4Males.assembled.fastq.gz,../all_samples/Group4Males.unassembled.forward.fastq.gz,../all_samples/Group4Males.unassembled.reverse.fastq.gz --bowtie2out Male4pear.bowtie2.bz2 --nproc 48 --input_type fastq -o pear_Male4.txt


merge_metaphlan_tables.py pear_Female3.txt pear_Male3.txt pear_Female4.txt pear_Male4.txt > metaphlanMERGED2.txt

sudo Rscript /home/drbrown/miniforge3/envs/mpa/lib/python3.7/site-packages/metaphlan/utils/calculate_diversity.R -f metaphlanMERGED2.txt -d beta -m bray-curtis

sudo Rscript /home/drbrown/miniforge3/envs/mpa/lib/python3.7/site-packages/metaphlan/utils/calculate_diversity.R -f metaphlanMERGED2.txt -d alpha -m shannon

grep -E "(s__)|(^ID)" metaphlanMERGED2.txt | grep -v "t__" | sed 's/^.*s__//g' > metaphlanMERGED2_species.txt

sgb_to_gtdb_profile.py -i metaphlanMERGED2.txt -o metaphlanMERGED2_gtdb.txt
```

paste the top two header rows of the metaphlanMERGED2.txt into the metaphlanMERGED2_species.txt file before doing the hclust steps below

hclust2.py -i metaphlanMERGED2_species.txt -o metaphlanMERGED2_species.sqrt_scale.png --ftop 215 -l --f_dist_f correlation --s_dist_f braycurtis --cell_aspect_ratio 0.1 -s --fperc 99 --colorbar_font_size 2 --slabel_size 5 --flabel_size 1 --legend_file metaphlanMERGED2_species.sqrt_scale.legend.png --max_flabel_len 100 --colormap bbcyr --metadata_height 0.015 --fdend_width 15 --sdend_height 1 --dpi 1000

changed parameters to get (1) families (2) species - with some refinement by changing the "stat" threshold from 0.2 default to 0.1, which should help to name a few more SGBs.
below is an example of the --tax_lev f

metaphlan ../all_samples/Group3Males.assembled.fastq,../all_samples/Group3Males.unassembled.forward.fastq,../all_samples/Group3Males.unassembled.reverse.fastq.gz --bowtie2out Male3fam.bowtie2.bz2 --tax_lev f --nproc 48 --input_type fastq -o fam_Male3.txt

below is an example of the --tax_lev s
to get species level only, but adding the --stat_q 0.1 to refine the taxa a bit more

metaphlan ../all_samples/Group3Males.assembled.fastq,../all_samples/Group3Males.unassembled.forward.fastq,../all_samples/Group3Males.unassembled.reverse.fastq.gz --bowtie2out Male3stat.bowtie2.bz2 --tax_lev s --stat_q 0.1 --nproc 48 --input_type fastq -o stat_Male3.txt

merge_metaphlan_tables.py fam_Female3.txt fam_Male3.txt fam_Female4.txt fam_Male4.txt > metaphlanMERGEDfam.txt

merge_metaphlan_tables.py stat_Female3.txt stat_Male3.txt stat_Female4.txt stat_Male4.txt > metaphlanMERGEDstat.txt

sed 's/s__//g' metaphlanMERGEDstat.txt > metaphlanMERGEDstatC.txt

The final family hclust2 plot

hclust2.py -i metaphlanMERGEDfamC.txt -o metaphlanMERGEDfam.sqrt_scale.png --ftop 215 -l --f_dist_f correlation --s_dist_f braycurtis --cell_aspect_ratio 0.4 -s --fperc 99 --colorbar_font_size 2 --slabel_size 7 --flabel_size 5 --legend_file metaphlanMERGEDfam.sqrt_scale.legend.png --max_flabel_len 100 --colormap bbcyr --metadata_height 0.015 --fdend_width 4 --sdend_height 1 --dpi 1000

The final species hclus2 plot

hclust2.py -i metaphlanMERGEDstatC.txt -o metaphlanMERGEDstat.sqrt_scale.png --ftop 215 -l --f_dist_f correlation --s_dist_f braycurtis --cell_aspect_ratio 0.06 -s --fperc 99 --colorbar_font_size 2 --slabel_size 7 --flabel_size 2 --legend_file metaphlanMERGEDstat.sqrt_scale.legend.png --max_flabel_len 100 --colormap bbcyr --metadata_height 0.015 --fdend_width 15 --sdend_height 1 --dpi 2000

CONVERT METAPHLAN4 OUTPUTS INTO PHYLOSEQ OBJECT FOR ANCOM-BC2:

make metaphlan output into phyloseq format (using a python script from flannsmith github site) then import into R to run ANCOM-BC

#use master "profile" outputs from metaphlan4 (called "pear_something")

imported each text file into excel, and used a concatenation (sorting and removing all but unique) for the full taxa columns, then used the leftmost column and left empty columns to fill with vlookup from each sample + abundance to produce one main file.

#then made the outputs into two files to compare factors using metaphlan_to_phyloseq_dfs.py from here
https://github.com/flannsmith/metaphlan-plot-by-taxa/blob/master/Converting%20Metaphlan%20profile%20to%20Phyloseq%20objects.ipynb
with some modifications: metaphlan_to_phyloseq_dfsMINE2.py

which generated

species_known_abundance.csv
species_known_taxa.csv
sample_df.csv

OTU columns were then moved from right to left before analysis in R

library(readxl)

#Used this tutorial
https://microbiome.github.io/OMA/differential-abundance.html

library(ANCOMBC)

library(magrittr)

#Phyloseq objects need to have row.names define the row names from the right columns

otu_mat<- read_excel("~/Desktop/PAPERS/GarrisonPaper/convert_metaphlan2phyloseq.xlsx", sheet = "outOTUspe")

tax_mat<- read_excel("~/Desktop/PAPERS/GarrisonPaper/convert_metaphlan2phyloseq.xlsx", sheet = "outTAXspe")

otu_mat <- otu_mat %>%
+ tibble::column_to_rownames("Otu")

tax_mat <- tax_mat %>%
+ tibble::column_to_rownames("Otu")

otu_mat <- as.matrix(otu_mat)

tax_mat <- as.matrix(tax_mat)

OTU = otu_table(otu_mat, taxa_are_rows = TRUE)

TAX = tax_table(tax_mat)

physpe <- phyloseq(OTU, TAX, samples)

tse = mia::makeTreeSummarizedExperimentFromPhyloseq(physpe)

RUN ANCOM-BC2:

ancombc2_out <- ancombc2(data = tse, fix_formula = "Sex + Group", p_adj_method = "fdr", prv_cut = 0, group = "Sex", struc_zero = TRUE, neg_lb = TRUE, tax_level = "Species", global = TRUE, verbose = TRUE, pairwise = TRUE, dunnet = TRUE, trend = TRUE)

PLOT NMDS BRAY ORDINATION:

physpe.ord <- ordinate(physpe, "NMDS", "bray")
plot_ordination(physpe, physpe.ord, type="taxa", color="Class", title="OTUs", label="Species") + facet_wrap(~Phylum, 3) + theme(legend.position="none")

plot_ordination(physpe, physpe.ord, type="split", color="Class", shape="Group", title="biplot", label = "Species") + geom_point(size=3)

library(vegan)

metadata <- as(sample_data(physpe), "data.frame")

adonis2(distance(physpe, method="bray") ~ Group, data = metadata)

PERMANOVA:

```
testDist <- physpe %>%
  tax_agg("Species") %>%
  tax_transform("identity") %>%
  dist_calc("bray")

PERM2 <- testDist %>%
  dist_permanova(
    seed = 1,
    variables = c("Sex", "Group"),
    n_processes = 1,
    n_perms = 99 # only 99 perms used in examples for speed (use 9999+!)
  )
```

METAPANGENOMIC ANALYSIS:

Metagenomic annotation with Prokka:

prokka --force --outdir GRP3Fprokka --locustag GRP3F --addgenes --evalue 0.001 --compliant /media/DataA/Emily/Assembled_Data/Scaffolds

Ortholog detection with Roary:

##moved all .gff files from prokka output into new directory, ran this line on these .gff files.

##change the numbers after “-f roary_____” to today’s date for easy finding later

roary -e -f roary05-21 --mafft -p 46 -i 60 -v *.gff

Gene ontology enrichment with TopGO:

##topGO code goes into R, make sure all packages are installed:
https://bioconductor.org/packages/devel/bioc/vignettes/topGO/inst/doc/topGO.pdf

```
#https://github.com/lyijin/topGO_pipeline/blob/master/aip_topgo_usage.consider_universe.R

# change this folder to point to your own "go_annot" folder
setwd('/Users/amanda/Desktop/SimranPaper1/topGObanana/go_annot')
library(topGO)

# remember to change the folder name to point to the folder containing your genes of interest lists
folder_of_interest = "./genes_of_interest/"

# exclude files with "universe" in it
mult_files = grep(list.files(folder_of_interest), pattern="*universe*", inv=T, value=T)

for (m in mult_files) {
  annot_filename = './go_annots.banana.tsv'
  gene_id_to_go = readMappings(file=annot_filename)

  # shrink list of all GO terms down to the correct universe
  universe_file = gsub('up', 'universe', m)
  universe_file = gsub('down', 'universe', universe_file)
  universe_file = gsub('diff', 'universe', universe_file)
  universe_genes = scan(paste0(folder_of_interest, universe_file), character(0), sep="\n")

  gene_id_to_go = gene_id_to_go[universe_genes]
  gene_id_to_go = gene_id_to_go[gene_id_to_go != 'no_hit']
  gene_names = names(gene_id_to_go)

  for (go_category in c('bp', 'cc', 'mf')) {
    print(paste("Current file:", m))
    genes_of_interest_filename = paste0(folder_of_interest, m)
    genes_of_interest = scan(genes_of_interest_filename, character(0), sep="\n")
```

```
    genelist = factor(as.integer(gene_names %in% genes_of_interest))
    names(genelist) = gene_names

    GOdata = try(new("topGOdata", ontology=toupper(go_category), allGenes=genelist,
gene2GO=gene_id_to_go, annotationFun=annFUN.gene2GO))

    # handle error
    if (class(GOdata) == "try-error") {
      print (paste0("Error for file", m, "!"))
      next
    }

    # weight01 is the default algorithm used in Alexa et al. (2006)
    weight01.fisher <- runTest(GOdata, statistic = "fisher")

    # generate a results table (for only the top 1000 GO terms)
    #   topNodes: highest 1000 GO terms shown
    #   numChar: truncates GO term descriptions at 1000 chars (basically, disables truncation)
    if (length(genes_of_interest) < 500) {
      results_table = GenTable(GOdata, P_value=weight01.fisher, orderBy="P_value", topNodes=100,
numChar=1000)
    } else {
      results_table = GenTable(GOdata, P_value=weight01.fisher, orderBy="P_value", topNodes=300,
numChar=1000)
    }

    # write it out into a file for python post-processing
    output_filename = paste0("./topGO_output/", go_category, "_", m)
    write.table(results_table, file=output_filename, quote=FALSE, sep='\t')
  }
}
```

##these outputs then can be summarized using:

```
#!/bin/bash

# remove previous summary files
rm -f summary_*.txt

# generate new summary files
# two criteria to pass:
# 1. At least 5 terms in the universe ($4)
# 2. P value < 0.05 OR if it contains the character '<' (because R outputs stuff like '< 1e-30') ($7)
for a in bp*.txt; do
    for b in $a ${a/bp/cc} ${a/bp/mf}; do
        echo -- $b -- >> ${a/bp_/summary_}
        awk -F $'\t' '{if ($4 >= 5 && ($7 < 0.05 || $7 ~ /^</)) print}' $b >> ${a/bp_/summary_}
```

```
    done
done

# prettify output
for a in summary_*.txt; do
    sed 's/GO\.ID/\tGO.ID/' $a | sed 's/-- /\n-- /' | sed 1d > tmp && mv -f tmp $a
done
```
