# Supplementary material for: Microbiome Taxonomic and Functional Differences in C3H/HeJ Mice Fed a Long-Term High-Fat Diet with Beef Protein ± Ammonium Hydroxide Supplementation
Source: Nutrients. 2024 May 25;16(11):1613. doi: 10.3390/nu16111613 (PMC11174526; doi:10.3390/nu16111613)
Supplement: Supplementary file 1 [file nutrients-16-01613-s001.zip › Supplementary Table S2.pdf]

Supplementary Table S2: Significantly enriched gene ontology (GO) categories from TopGO analysis for comparisons between supplemented (HFBN) and unsupplemented (HFB) high-fat beef diet microbiomes and males and females.

GO_ID	Term	Annot-ated	Signif-icant	Expected	P-value
**Enriched in supplemented HFBN versus unsupplemented HFB:**					
**Biological Processes:**					
GO:0031460	glycine betaine transport	10	3	0.35	0.0041
GO:1990961	xenobiotic detoxification by transmembrane export across the plasma membrane	13	3	0.45	0.0091
GO:0042742	defense response to bacterium	17	3	0.59	0.0196
GO:0042128	nitrate assimilation	9	2	0.31	0.0369
GO:0009061	anaerobic respiration	9	2	0.31	0.0369
**Cellular Components:**					
GO:0016021	integral component of membrane	1110	53	43.29	0.019
**Metabolic Functions:**					
GO:0000155	phosphorelay sensor kinase activity	53	8	1.98	0.00068
GO:0031177	phosphopantetheine binding	12	3	0.45	0.00882
GO:0031405	lipoic acid binding	5	2	0.19	0.01292
GO:0003824	catalytic activity	2844	107	106.43	0.03005
GO:0090729	toxin activity	8	2	0.3	0.03359
GO:0004674	protein serine/threonine kinase activity	8	2	0.3	0.03359
**Enriched in unsupplemented HFB versus supplemented HFBN:**					
**Biological Processes:**					
GO:0006749	glutathione metabolic process	10	4	0.73	0.0041
GO:0006979	response to oxidative stress	32	8	2.34	0.0075
GO:0006811	ion transport	194	19	14.17	0.0187
GO:0022904	respiratory electron transport chain	56	9	4.09	0.0333
GO:0019439	aromatic compound catabolic process	121	17	8.84	0.0429
GO:0019634	organic phosphonate metabolic process	9	4	0.66	0.0453
GO:0015774	polysaccharide transport	5	2	0.37	0.0459
GO:0005985	sucrose metabolic process	5	2	0.37	0.0459
GO:0022611	dormancy process	5	2	0.37	0.0459
**Cellular Components:**					
GO:0046930	pore complex	7	4	0.52	0.00089
GO:0009279	cell outer membrane	51	12	3.81	0.00106
GO:0042597	periplasmic space	125	18	9.35	0.00347
GO:0043190	ATP-binding cassette (ABC) transporter complex	51	9	3.81	0.0117
GO:0016021	integral component of membrane	1110	102	83.02	0.01534
**Metabolic Functions:**					
GO:0022857	transmembrane transporter activity	575	62	42.48	0.0023
GO:0004364	glutathione transferase activity	5	3	0.37	0.0036
GO:0015288	porin activity	8	5	0.59	0.0066
GO:0005507	copper ion binding	14	4	1.03	0.0162
GO:0005351	carbohydrate:proton symporter activity	9	3	0.66	0.024
GO:0005355	glucose transmembrane transporter activity	5	2	0.37	0.0469
GO:0050660	flavin adenine dinucleotide binding	68	10	5.02	0.0486
**Enriched in Females versus Males:**					
**Biological Processes:**					
GO:0009636	response to toxic substance	40	5	1.24	0.012
GO:0019646	aerobic electron transport chain	6	2	0.19	0.013
GO:0042128	nitrate assimilation	9	3	0.28	0.023

Supplementary Table S2: Significantly enriched gene ontology (GO) categories from TopGO analysis for comparisons between supplemented (HFBN) and unsupplemented (HFB) high-fat beef diet microbiomes and males and females.

GO:0016117	carotenoid biosynthetic process	8	2	0.25	0.024
GO:0042168	heme metabolic process	13	2	0.4	0.031
**Metabolic Functions:**					
GO:0071949	FAD binding	22	3	0.71	0.032
GO:0005504	fatty acid binding	6	2	0.19	0.032
GO:0008745	N-acetylmuramoyl-L-alanine amidase activity	10	2	0.32	0.039
**Enriched in Males versus Females:**					
**Biological Processes:**					
GO:0006749	glutathione metabolic process	10	4	0.72	0.004
GO:0006811	ion transport	189	18	13.67	0.018
GO:0031460	glycine betaine transport	10	3	0.72	0.031
GO:0006979	response to oxidative stress	32	6	2.31	0.036
GO:0019634	organic phosphonate metabolic process	9	4	0.65	0.044
GO:0015774	polysaccharide transport	5	2	0.36	0.045
GO:0022611	dormancy process	5	2	0.36	0.045
GO:0007155	cell adhesion	22	6	1.59	0.049
**Cellular Components:**					
GO:0046930	pore complex	7	4	0.53	0.00091
GO:0009279	cell outer membrane	51	11	3.84	0.00425
GO:0042597	periplasmic space	125	17	9.41	0.01042
GO:0016021	integral component of membrane	1110	100	83.53	0.0165
GO:0019867	outer membrane	56	13	4.21	0.04496
**Metabolic Functions:**					
GO:0031177	phosphopantetheine binding	12	5	0.88	0.0011
GO:0004364	glutathione transferase activity	5	3	0.37	0.0035
GO:0036424	L-phosphoserine phosphatase activity	5	3	0.37	0.0035
GO:0015288	porin activity	8	5	0.59	0.0065
GO:0050660	flavin adenine dinucleotide binding	68	11	4.99	0.0168
GO:0004315	3-oxoacyl-[acyl-carrier-protein] synthase activity	9	3	0.66	0.0236
GO:0000155	phosphorelay sensor kinase activity	53	8	3.89	0.0375
GO:0043856	anti-sigma factor antagonist activity	5	2	0.37	0.0463
