# Supplementary material for: Microbiome Taxonomic and Functional Differences in C3H/HeJ Mice Fed a Long-Term High-Fat Diet with Beef Protein ± Ammonium Hydroxide Supplementation
Source: Nutrients. 2024 May 25;16(11):1613. doi: 10.3390/nu16111613 (PMC11174526; doi:10.3390/nu16111613)
Supplement: Supplementary file 1 [file nutrients-16-01613-s001.zip › Supplementary Table S3.pdf]

Supplementary Table S3: Gene ontology (GO) categories plotted in GO-Figure for numbered categories shown in circles in Figure 8

Diet pH Treated (HFBN) Biological Process

1. positive regulation of cell population proliferati...
2. glycine betaine transport
3. L-fucose catabolic process
4. xenobiotic detoxification by transmembrane export ...
5. defense response to bacterium
6. alkane catabolic process
7. homocysteine catabolic process
8. ciliary neurotrophic factor-mediated signaling pat...
9. cellular response to magnesium starvation
10. muscle organ morphogenesis
11. cellular response to nitrite
12. positive regulation of axon regeneration
13. regulation of retinal cell programmed cell death
14. astrocyte activation
15. detoxification of mercury ion
16. heparan sulfate proteoglycan catabolic process
17. protein ADP-ribosylation
18. peptidyl-threonine phosphorylation
19. negative regulation of photoreceptor cell differen...
20. negative regulation of protein catabolic process

Diet pH Treated (HFBN) Molecular Function

1. phosphorelay sensor kinase activity
2. mercury ion binding
3. dihydrolipoyllysine-residue (2-methylpropanoyl)tra...
4. phosphopantetheine binding
5. lipoic acid binding
6. catalytic activity
7. toxin activity
8. 2-methyleneglutarate mutase activity
9. L-galactose dehydrogenase activity
10. chloride peroxidase activity
11. heparin-sulfate lyase activity
12. pyrroline-2-carboxylate reductase activity
13. pyroglutamyl-peptidase activity
14. mannosylfructose-phosphate synthase activity
15. benzoylformate decarboxylase activity
16. molybdenum ion transmembrane transporter activity
17. complement component C3b binding
18. homocysteine desulfhydrase activity
19. N-isopropylammelide isopropylaminohydrolase activi...
20. delta1-piperideine-2-carboxylate reductase activit...

Control Diet (HFB) Biological Process

1. glutathione metabolic process
2. response to oxidative stress
3. toxin transport
4. fatty acid elongation
5. organic phosphonate catabolic process
6. respiratory electron transport chain
7. polysaccharide transport
8. sucrose metabolic process
9. dormancy process
10. cellular aromatic compound metabolic process
11. type IV pilus-dependent motility
12. 2,4,6-trinitrotoluene catabolic process
13. taurine transport
14. 2,4,5-trichlorophenoxyacetic acid catabolic proces...
15. arsenite transport
16. allantoin assimilation pathway
17. cellular response to glucose-phosphate stress
18. peptidyl-arginine hydroxylation
19. response to DDT
20. regulation of nitrate assimilation

Control Diet (HFB) Molecular Function

1. transmembrane transporter activity
2. glutathione transferase activity
3. 4-formylbenzenesulfonate dehydrogenase activity
4. maltose alpha-glucosidase activity
5. porin activity
6. copper ion binding
7. carbohydrate:proton symporter activity
8. ribonucleoside-diphosphate reductase activity, thi...
9. flavin adenine dinucleotide binding
10. NADP binding
11. minor groove of adenine-thymine-rich DNA binding
12. oleate hydratase activity
13. S-methyl-5-thioadenosine phosphorylase activity
14. methenyltetrahydromethanopterin cyclohydrolase act...
15. arylamine N-acetyltransferase activity
16. 1-deoxy-D-xylulose kinase activity
17. dimethylhistidine N-methyltransferase activity
18. p-benzoquinone reductase (NADPH) activity
19. NAD(P)H nitroreductase activity
20. 2-aminomuconate deaminase activity

Female Biological Process

1. response to toxic substance
2. aerobic electron transport chain
3. carotenoid biosynthetic process
4. S-methylmethionine cycle
5. 2,4,6-trinitrotoluene catabolic process
6. Mo(VI)-molybdopterin cytosine dinucleotide biosynt...
7. glycolate catabolic process
8. protein ADP-ribosylation
9. inositol biosynthetic process
10. detection of visible light
11. taurine transport
12. regulation of nitrate assimilation
13. homocysteine catabolic process
14. oxaloacetate metabolic process
15. electron transport coupled proton transport
16. type IV pilus-dependent motility
17. denitrification pathway
18. heme A biosynthetic process
19. heparan sulfate proteoglycan catabolic process
20. pyochelin biosynthetic process

Female Molecular Function

1. mercury ion binding
2. cytochrome-c oxidase activity
3. oxidoreductase activity, acting on NAD(P)H, heme p...
4. N-isopropylammelide isopropylaminohydrolase activi...
5. molybdenum cofactor cytidylyltransferase activity
6. homocysteine desulfhydrase activity
7. hydantoin racemase activity
8. delta1-piperideine-2-carboxylate reductase activit...
9. pyroglutamyl-peptidase activity
10. pyrroline-2-carboxylate reductase activity
11. heparin-sulfate lyase activity
12. 5'-deoxyribose-5-phosphate lyase activity
13. L-galactose dehydrogenase activity
14. oleate hydratase activity
15. exodeoxyribonuclease I activity
16. kynurenine 3-monooxygenase activity
17. hydroxyisourate hydrolase activity
18. S-adenosylmethionine-homocysteine S-methyltransfer...
19. FAD binding
20. fatty acid binding

Male Biological Process

1. glutathione metabolic process
2. cellulose biosynthetic process
3. beta-ketoadipate pathway
4. organic phosphonate catabolic process
5. glycine betaine transport
6. response to oxidative stress
7. polysaccharide transport
8. dormancy process
9. cell adhesion
10. respiratory electron transport chain
11. 2,4,5-trichlorophenoxyacetic acid catabolic proces...
12. arsenite transport
13. negative regulation of neuron apoptotic process
14. peptidyl-arginine hydroxylation
15. response to DDT
16. positive regulation of cell growth
17. detoxification of mercury ion
18. cellular response to glucose-phosphate stress
19. positive regulation of tyrosine phosphorylation of...
20. iron ion transmembrane transport

Male Molecular Function

1. phosphopantetheine binding
2. glutathione transferase activity
3. L-phosphoserine phosphatase activity
4. porin activity
5. flavin adenine dinucleotide binding
6. 3-oxoacyl-[acyl-carrier-protein] synthase activity
7. phosphorelay sensor kinase activity
8. anti-sigma factor antagonist activity
9. 2-aminomuconate deaminase activity
10. p-benzoquinone reductase (NADPH) activity
11. isocitrate lyase activity
12. aconitate delta-isomerase activity
13. DNA/RNA helicase activity
14. minor groove of adenine-thymine-rich DNA binding
15. citrate:proton symporter activity
16. 3,4-dihydroxyphenylacetate 2,3-dioxygenase activit...
17. complement component C3b binding
18. oxidized pyrimidine nucleobase lesion DNA N-glycos...
19. antimonite transmembrane transporter activity
20. mercury (II) reductase activity
